# Characterization of international migration movements toward Chile: A scoping review of scientific articles and official reports

**DOI:** 10.1016/j.jmh.2025.100363

**Published:** 2025-09-21

**Authors:** María Belén Reinoso-Cataldo, Valeria Stuardo, Cecilia Bustos-Ibarra, Julieta Belmar, Cristian Lisboa, Kenny Low, Sonia Parella Rubio, Constanza Adrian Parra, Mercedes Carrasco-Portiño

**Affiliations:** a Master’s Program in Sexual and Reproductive Health, Department of Obstetrics and Childcare, Faculty of Medicine, University of Concepción. Concepción, Chile; b Public Health Institute, University Andrés Bello, Chile; c Department of Social Work, Faculty of Social Sciences, University of Concepción, Chile; d Epidemiology Program, School of Public Health, Faculty of Medicine, University of Chile, Chile; e School of Dentistry, Faculty of Health Sciences, Autonomous University of Chile, Chile; f Independent Researcher, Chile; g CER-Migracions, Department of Sociology, Autonomous University of Barcelona, Spain; h PhD in Biomedical Research Methodology and Public Health, Department of Pediatrics, Obstetrics and Gynecology and Preventive Medicine and Public Health, Autonomous University of Barcelona, Spain; i Department of Obstetrics and Childcare, Faculty of Medicine, University of Concepción, Chile

**Keywords:** Transients and migrants, Emigration and immigration, Chile

## Abstract

In the last decade, Chile has emerged as a receiving country for migrants from Latin America and the Caribbean (LAC). The scale of this phenomenon has sparked increasing interest in understanding its impact on various sectors such as healthcare and education. This study aims to characterize the scientific evidence and official reports on international migration toward Chile from 1990 to 2024. A scoping review was conducted. Global inclusion criteria: These encompassed articles and official reports published between 1990 and 2024 focusing on the migrant population toward Chile. Databases for the articles: These included PubMed, Scopus, WoS, and SciELO. Keywords: These included Transients and Migrants, Emigration and Immigration, Population Dynamics, Human Migration, Chile, South America, Latin America, and Freedom of Movement. Sources for the official reports: These included National Statistics Institute (INE for its acronym in Spanish); Department of Immigration and Foreign Services; Jesuit Migrant Service; National Human Rights Institute (INDH for its acronym in Spanish); and The UN Refugee Agency (UNHCR). No keywords were used. Global variables: These included type of study, sociodemographic characteristics, type of migration, object of study, main results, limitations, and conclusions. A concordance test of the questionnaire was conducted for the articles and official reports, yielding 91 % and 94 % agreement between observers, respectively.

Accordingly, 21 articles and 28 official reports were included. In both types of sources, the study population included the entire life cycle, primarily from countries in LAC. The observed types of migration included international (voluntary, forced, or humanitarian). Articles focused on measuring mental health (MH) and the access/use of healthcare services. Regarding MH, it was observed that the young migrant population exhibited worse indicators than the adult population, while both migrant and Chilean populations exhibited similar MH statistics, with socioeconomic level (SEL) being a significant determinant. Access to healthcare services has increased among the migrant population and is contingent upon SEL. Only two articles have addressed subjects related to reproductive health, with none discussing sexual health. Official reports focused on characterizing the population and their access to services (healthcare, education, housing, occupational situation), border mobility, poverty index, social perceptions, and inclusion. Most studies have utilized secondary data provided by official sources.

Migration toward Chile primarily involves south–south migration, sociodemographic characterization, and issues accessing services, including healthcare. Results highlight a scarcity of studies collecting primary data, leading to a lack of relevant indicators for understanding aspects such as migration causes, attracting factors, migration trajectory, migration status, cross-cultural relationships, or sexual health.

## 1 Introduction

### 1.1 Background

In the last two decades, international migration toward Chile has become relevant. The country has transformed into one of the primary destinations for South American migration. Different from previous migration flows, which predominantly originated from Europe, current migration trends have a regional origin (Canales, 2019). After 2000 the largest national groups migrating to Chile were from Peru, Bolivia, and Colombia (Stefoni, 2011), with a significant female presence. Lately, countries that have contributed to this migration flow include the Dominican Republic, Haiti, and Venezuela. (Canales, 2019).

Undoubtedly, a portion of this recent flow toward Chile has to do with the so called involuntary or forced mobility, as well as hopelessness (Castles, 2003). These migrations, characterized by precariousness, imply scenarios highly susceptible to the violation of rights. Forced migration results from sociological categories that should be expressed not solely through demarcation criteria, but rather by considering long-term continuums. Therefore, forced migration cannot be addressed from the traditional legal definitions that differentiate refugee persons from the rest, considering them economic migrants that move voluntarily and autonomously, based on a completely economic premise (Parella, 2022). Such is the case of countries like Haiti, struck by environmental and natural disasters, or Venezuela, with its forced migration that has intensified since 2015 because of the humanitarian emergency that the country faces (Stefoni, 2019).

Some factors that contribute to shaping the migration dynamics toward Chile are the following: The political stability and the economic growth after the recovery of democracy; the European economic crisis of 2008 and the subsequent return and re-emigration processes; poverty levels and the informal labor market of some of the countries of origin, especially considering the economic stagnation experienced by countries like Argentina and specifically Venezuela, which were the main destination places for South American migration historically (Canales, 2019); the tough migration policies in more developed economies, like those of the United States; the closeness of Chile, resulting in less travel cost in the context of regional migration and the support networks that have been developed throughout the years that allow and/or facilitate migrants who already live in the country to receive family members, friends, and acquaintances (Stefoni, 2011).

In December 2021, the estimations revealed that 1482,390 foreign persons were regular residents. Contrary to the flow of the 1990s and 2000s, only one country shared a border with Chile among the predominant nationalities among the foreign population in 2021. Accordingly, 30 % were from Venezuela, 16.6 % from Peru, 12.2 % from Haiti, and 11.7 % from Colombia (National Migration Service, 2023).

The 2018 estimations indicated a population of 182,958 individuals, revealing an absolute growth and a relative growth of 14.1 % for the same period. The 2020 estimations revealed an absolute growth of 22,343 people and a relative growth of 1.5 % (National Statistics Institute, 2022).

The data revealed that 10.8 % of migrants live in socioeconomic vulnerability, earning an income below the poverty line, consistent with the statistics for people born in Chile. Regardless, both groups exhibited a constant decrease in income poverty since 2009. Despite the cross-sectional decrease in poverty, the gap between the two groups has been constantly reduced and was even reversed in 2017. Since 2006, migrants had lower levels of income poverty compared with people born in Chile. This increasing relative incidence of poverty among migrants is a clear sign that should be considered (Irarrázaval, 2019).

Multidimensional poverty, which considers variables such as healthcare, education, housing, work, and social security, indicates differences between the native population and people born outside the country. While 20.5 % of people from Chile are in a situation of multidimensional poverty, migrants represent 24.6 % of this category. Since income poverty does not indicate relevant differences, it can be inferred that the main nuances are access to healthcare, education, housing, and labor conditions (Irarrázaval, 2019).

The situation of vulnerability that primarily affects the migrant population and has several causes must be analyzed from the perspective of the destination country. It is produced during the different stages of the migration cycle: origin, transit, and destination, and it is the result of the intersection of different focal points of their condition of inequality, such as gender, social class, ethnic/racial issues, age, etc. (Maldonado et al., 2018). Therefore, during the migration process, people experience contexts of mobility under circumstances that may affect their physical and mental well-being (Urzúa, 2016). Certainly, migrants often face poor health results in the countries of transit and destination due to multiple challenges they undergo, such as language and cultural differences, discrimination, restricted access to healthcare services, poverty, lack of housing, and exploitation (World Health Organization, 2022).

Thus, it is crucial to have data on the migrant population that include the complete migration process and that can be used to assess and design comprehensive healthcare policies that are accessible and can be adapted to the cultural and socioeconomic specificities. It is about providing the migrant population in a situation of vulnerability, regardless of their migration status, the necessary healthcare support, providing identification and assistance, and ensuring the protection of their human rights.

### 1.2 Objective

A scoping review was conducted to characterize the scientific evidence and official reports on international migration toward Chile during 1990–2024 to systematize the available national data on migration processes during the period previously mentioned. The research question that needs to be answered is: what are the characteristics of international migration processes to Chile in the period 1990–2024?

## 2 Methods

### 2.1 Scoping review approach

This scoping review is based on methodological frameworks by Arksey and O´Malley (steps of a scoping review) (Arksey and O´Malley, 2005) and the Johanna Briggs Institute (JBI, Population/Concept/Context (PCC) framework) (Peters et al., 2024). We report according to the recommendations of the Preferred Reporting Items for Systematic reviews and Meta-Analysis extension for Scoping Reviews (PRISMA-ScR) (Tricco et al., 2018). The PRISMA-ScRT Checklist is provided in the supplementary material (Appendix A). A protocol has been registered prior to accessing the data via OSF (https://osf.io/uazf7)

This scoping review had two stages: The first stage included a data search in scientific databases to obtain empirical articles. The second stage was based on obtaining official reports from national and international organizations (grey literature).

### 2.2 Search strategy

**First stage: Data search in scientific databases**

The **databases** used included PubMed, Scopus, Web of Science, and SciELO. **The keywords used in the scientific databases** included Transients and Migrants (MeSH); Emigration and Immigration (MeSH, introduced in 1963); Population Dynamics (MeSH, introduced in 1976); Human Migration (MeSH, introduced in 2013); Chile (MeSH); and Freedom of Movement (MeSH, introduced in 2021). In the Scielo database, the controlled terms (Descriptores de Ciencias de la Salud (DECS)) were used in Spanish and were “migrants and Chile”. Details on search strings of each database are provided in Appendix B.

**Second stage: Review of official reports from national and international organizations (Grey literature)**

The **databases** used included the National Statistics Institute; National Migration Service; Jesuit Migrant Service; National Human Rights Institute; and the United Nations Refugee Agency (UNHCR). The details of each source, including the platforms, links and search instructions, are provided in Appendix C.

### 2.3 Elegibility criteria

**First stage: Scientific articles**

The **inclusion criteria** encompassed articles with an available abstract that research the characteristics of migration processes toward Chile between 1990 and 2024. Articles written in Spanish, English, Portuguese, and French were considered, as well as original empirical studies.

**Second stage: Official reports from national and international organizations (Grey literature)**

The **inclusion criteria** included reports examining the characteristics of migration processes toward Chile between 1990 and 2024, published in Spanish, English, Portuguese, and French, as well as empirical studies.

### 2.4 Study selection

**First stage: Scientific articles**

Potentially relevant articles were screened at title/abstract level and full-text level using EndNote by three investigators (MBRC; MCP; CBI).

**Second stage: Official reports from national and international organizations (Grey literature)**

Regarding the search in the grey literature, the websites of the selected sources were accessed. Each of these has a different search pattern, each of which has a section where their studies and publications can be found, which were selected according to eligibility criteria: empirical study, in Spanish or English, from 1990 to 2024, focused on migration in Chile. This search and selection were carried out by a researcher (MBRC).

### 2.5 Data extraction and synthesis

For the **data collection** of the scientific documents (articles and officially reports), 87 variables were created, which included extrinsic variables, encompassing general characteristics of the publications such as the object of study and publication year, among others. In addition, methodological variables were added, such as type of study, data collection technique, and sample size, among others. Lastly, substantive variables related to the population under study were included, along with other results related to the migration process, such as type of migration, causes of migration, migration trajectory, attracting factors, and acculturation.

To accommodate differences in the structure of scientific articles (see Appendix D) and official reports (see Appendix E), two **instruments** for data collection were created for each type of publication. The first instrument included 44 questions, and the second one contained 36 questions. Concordance tests were conducted between the three observers, resulting in 91 % and 94 % concordance, respectively, indicating good concordance.

A data extraction matrix was developed in Microsoft Excel. Original data (text paragraphs) were extracted from full-texts by three researchers (MBRC, MCP, CBI) into the matrix. Next, tables were developed in Microsoft Word to synthetize data into major topics. First, the general characteristics of the publications were identified (see Table 1). Secondly, the methodological approaches used to study the migration processes to Chile in the period are described (see Table 2). Thirdly, the bio-socio-demographic profile of the population under study is described (see Table 3). Finally, the approach to migration is described in official articles and reports up to 2024 (see Table 4).

**Table 1:** Identification of the general characteristics of scientific publications and official reports.

SCIENTIFIC ARTICLES				
1st author (year)		Country of 1st author*	Journal	Object of study
Parson, 2010		United States	Violence Against Women	Intersectionality, gender inequalities, violence, migration. Anthropological case study.
Rojas, 2011		Chile	Chile’s Medical Journal	Prevalence of Common Mental Disorders in Migrant Population and Access to Mental Health
Cabieses, 2012a		Chile	International Journal for Equity in Health	Access and use of health services migrant population vs. national population
Cabieses, 2012b		Chile	International Journal of Environmental Research and Public Health	Disability prevalence and associated factors migrant vs. national population
Cabieses, 2013		Chile	Chile’s Medical Journal	Contrasting the “Latin paradox” migrant v/s national population
Cabieses, 2017		Chile	Chile’s Pediatrics Journal	Gaps in health outcomes in migrant children
Bernales, 2018		Chile	Mexico’s Public Health	Social Health Determinants of migrant children from the perspective of key actors
Grande, 2019		Spain	International Migrations	Relationship between fertility and migration/reproductive patterns in Peruvian migrants in Chile and Spain
Carreño, 2020		Chile	Collective Health	Mental health needs of refugees and asylum seekers
Caqueo-Urízar, 2021		Chile	Journal of Immigrant and Minority Health	Effects of migration on the mental health of migrant/national children and adolescents
Segovia, 2021		Chile	Int Journal of Environmental Research and Public Health	Social-labor integration experiences of Colombian migrants
Roessler, 2021a		Chile	Clinical and Social Medicine	Relationship between a set of sociodemographic and economic variables with the implementation of quarantine in the Venezuelan population residing in Chile
Chen, 2022		USA	Int Journal of Environmental Research and Public Health	Migrant experiences and COVID-19 impact on the mental health of women from Haiti
Margarit, 2022a		Chile	Rumbos TS	Problematize mobility in the dimension of the daily life of migrant women in the commune of Santiago and understand how they move and build a new migrant spatiality based on their daily journeys.
Margarit, 2022b		Chile	INVI Journal	Problematize migrant living through the dimensions of housing, home, and location of migrants living in Greater Santiago.
Barahona, 2022		Spain	Rumbos TS	Establish the macroeconomic variables associated with Venezuelan migration in Chile for 2010–2020.
Blukacz, 2023		Chile	Revista Da Escola de Enfermagem	Experience and perception of access to health services during the pandemic.
Iribarne-Wiff, 2023		Chile	Revista Salud Pública de México	Health rights migrants in the normative and programmatic framework and its relationship with health outcomes during the COVID19 pandemic.
Baeza-Rivera, 2023		Chile	Revista Médica de Chile	Health workers satisfaction and aspects of health care improvement.
Castillo Lobos, 2023		Chile	Revista Salud Colectiva	Migratory grief, loss of ties and origin distance, expectations vs. reality and overstrain validation.
Oyarte, 2023		Chile	International Journal of Environmental Research and Public Health	Access to and use of health care services among international migrants.
OFFICIAL REPORTS				
1st authorship (year)	Institution that publishes the report		Participant institution/s	Object of study
Desplanque, 2012	National Institute of Human Rights		University Academy of Christian Humanism	Sociocultural insertion process of a group of Palestinian refugee families in Chile.
Rojas, 2014	Jesuit Migrant Service Foundation		International Migration Organization	Quality of work to which the migrant population has access and, on the other hand, analyzes the factors that operate in their labor inclusion or exclusion.
International Migration Organization Chile, 2015	National Institute of Human Rights		IOM Chile, Municipality of Quilicura, UNHCR, U Catholic, U Chile, U central, USACH, CESC U. Chile	Characteristics of the international migrant population living in the community of Quilicura in relation to their current living conditions, migratory situation, and social relations.
Vicuña, 2015	National Institute of Human Rights		Jesuit Migrant Service	Understand and generate visibility of a reality that has historically occurred in the area, with the use of statistical data and technical and reflective contributions.
Ministry of the Interior and Public Security, 2016	Department of Immigration and Foreign Services of the Ministry of the Interior and Public Security.		——–	Generate a relevant source of consultation regarding what happened in our country in terms of migration, nationalization, and refuge. Acknowledge the development and characteristics that these phenomena have acquired in this last decade in Chile.
Troncoso, 2018	Jesuit Migrant Service		COLUNGA Foundation	Assessment of the migrant housing situation focused on Antofagasta and some communes in the metropolitan area of ​​Santiago (Quilicura, Estación Central, Santiago, Independencia, Recoleta, Renca).
National Institute of Statistics, 2018	National Institute of Statistics		INE: Department of demography and census, sub-department of demography and vitals	Characteristics of the international immigrant population living in Chile using the 2017 Population and Housing Census as a source of information
Jesuit Migrant Service, 2019	Jesuit Migrant Service		EKHOS Agency	Learn about the opinions and expectations of migrants living in Chile.
Brondi, 2019	Jesuit Migrant Service		———————-	The situation of migrants (mainly Venezuelans) after the entry into force of the new measures for entry into Chile.
Roessler, 2019	Jesuit Migrant Service		AVINA Foundation	Characteristics of the situation of the migrant population in Chile in 2019 in migration status, economy, and work, education, housing, inclusion, and security.
National Institute of Statistics, 2019	National Institute of Statistics		—————–	International movement of passengers according to records from the PDI border control databases in 2019.
Roessler, 2020a	Jesuit Migrant Service		Home of Christ, Center for Ethics and Social Reflection UAH	Present critical points of access to formal education for migrant children, adolescents, and their families.
Roessler, 2020b	Jesuit Migrant Service		TECHO, Department of Sociology U Chile, Center for Ethics and Social Reflection UAH	Characteristics of access to housing for people who migrate toward Chile.
Vicuña, 2020	Jesuit Migrant Service		——————-	Present certain dynamics of border mobilities that have occurred in the extreme north of Chile in 2020, focusing especially on the Regions of Arica and Parinacota (hereinafter RAandP) and Tarapacá (RT).
Cabieses, 2020	Jesuit Migrant Service		Institute of Sciences and Innovation in Medicine, Faculty of Medicine, German Clinic, University of Development, (ICIM for its acronym in Spanish), Medical College of Chile, MICROB-R	Knowledge of international migrant populations living in Chile about COVID-19 and its prevention measures.
National Institute of Statistics, 2020	National Institute of Statistics		——————-	International movement of passengers according to records from the PDI border control databases in 2020.
Roessler, 2021b	Jesuit Migrant Service		——————-	Understand what elements associated with work, social security, and institutional networks could affect poverty rates in the migrant population to a greater extent than the Chilean population.
Roessler, 2021c	Jesuit Migrant Service		——————-	Characteristics of access to school and higher education of the migrant population, identifying possible gaps related to the local community; understanding the role of adult migrant population’s education level in relation to the socio-labor opportunities that they actually find in Chile.
Jesuit Migrant Service, 2021	Jesuit Migrant Service		EKHOS Agency	Learn about the opinions and expectations of migrants living in Chile, their quality of life, and various social, economic, and political topics.
Roessler, 2021d	Jesuit Migrant Service		Antonio Ruiz de Montoya University, Konrad-Adenauer-Stiftung e.V., OBIMID	Present the current employment situation of the migrant population in Chile and emphasize proposals that address the critical points of said assessment.
Roessler, 2022	Jesuit Migrant Service		Medical College of Chile, Program of Social Studies in Health, Institute of Sciences and Innovation in Medicine (ICIM)	Characterize access to the Chilean health system of the migrant population, identifying possible gaps in relation to the local community.
Zúñiga, 2022	Jesuit Migrant Service		——————	Analyze the factors that influence and characterize the occupational situation of migrant women in Chile in 2020.
Migration and human mobility observatory, 2022	Jesuit Migrant Service		Migration and human mobility observatory	Knowledge and perceptions of the migrant and refugee population living in Chile on relevant aspects of the entry into force of the new Immigration and Foreign Services Law (21,325).
World Bank, 2022	Department of Immigration and Foreign Services of the Ministry of the Interior and Public Security.		World Bank, Joint Data Center of Forced Displacement, Migrations Chile, UC Center: surveys and longitudinal studies	Characteristics of the populations that have arrived in the country in the last five years, as well as the barriers to their integration, which serve as input to support the design of public policies for migration management in the country.
Jesuit Migrants Service, 2024a	Jesuit Migrant Service Advocacy and Studies Area.		———————–	Human mobility in Chile during 2023 and its evolution in recent years, with a review of migratory flows, the protection of human rights of migrants and refugees, as well as the status of their labor insertion and exercise of rights.
Jesuit Migrants Service, 2024b	Jesuit Migrant Service Advocacy and Studies Area.		UNHCR-ACNUR	Characteristics of human mobility flows in Chile, and the recognition of international protection needs in Chile in the period 2022–2023.
National Migration Service, 2024a	Studies Department of the National Migration Service.		———————–	Trends characteristics of administrative records between 2014 and 2023, looking for sociodemographic transformations in the migration pattern, and operability key elements of the institutional migration framework.
National Migration Service, 2024b	———–		———————–	Residence permits characteristics provided between January-December 2023, based on a gender approach.

**Table 2:** Identification of the methodological approach in which the migration processes toward Chile were studied in the period.

SCIENTIFIC ARTICLES			
1st authorship (year)	Methodology	Design/Technique	Type of data (Source)
Parson, 2010	Qualitative	Life story	Primary
Rojas, 2011	Quantitative	Cross-sectional	Primary
Cabieses, 2012a	Quantitative	Cross-sectional	Primary
Cabieses, 2012b	Quantitative	Cross-sectional	Secondary (CASEN 2006 and 2009)
Cabieses, 2013	Quantitative	Cross-sectional	Secondary (CASEN 2006)
Cabieses, 2017	Quantitative	Cross-sectional	Secondary (Chile Grows with you, CASEN 2013, Hospital Discharges 2012 DEIS)
Bernales, 2018	Qualitative	Interview and focus group	Primary
Grande, 2019	Quantitative	Cross-sectional	Secondary (Chilean Birth Registry, 2012 Population and Housing Census of Chile, Continuous Municipal Register of 2007 and 2008, (Spain), and the Natural Movement of the Population of 2007 (Spain and Population Census of Peru)
Carreño, 2020	Qualitative	Exploratory descriptive	Primary
Caqueo-Urízar, 2021	Quantitative	Cross-sectional	Primary
Segovia, 2021	Qualitative	Interview and focus group	Primary
Roessler, 2021a	Quantitative	Cross-sectional	Primary
Chen, 2022	Quantitative	Cross-sectional	Primary
Margarit, 2022a	Quantitative	Cross-sectional	Secondary (Research from the University of Santiago de Chile; Research from the Millennium Nucleus Mobilities and Territories)
Margarit, 2022b	Quantitative	Cross-sectional	Secondary (2017 Population and Housing Census of Chile and results of the Migrant Voices Survey (Jesuit Migrant Service, 2019).
Barahona, 2022	Quantitative	Cross-sectional	Secondary (Department of Immigration and Foreign Services of the Ministry of the Interior and Public Security.)
Blukacz, 2023	Qualitative	Interview	Primary
Iribarne-Wiff, 2023	Mixed (qualitative and quantitative)	Documentary analysis, cross-sectional	Secondary (Qualitative: technical standards, policies, technical guidance and official communications) (Quantitative: Hospital discharges 2014 to 2021; CASEN 2015; 2017 and 2020)
Baeza-Rivera, 2023	Quantitative	Cross-sectional psychometric	Primary
Castillo Lobos, 2023	Qualitative	Interview	Primary
Oyarte, 2023	Quantitative	Cross-sectional	Secondary (CASEN 2011–2017)
OFFICIAL REPORTS			
1st authorship (year)	Methodology	Design/Technique	Type of data (Source)
Desplanque, 2012	Qualitative	Exploratory Study/In-Depth Interview	Primary
Rojas, 2014	Quantitative	Cross-sectional	Primary
International Migration Organization Chile, 2015	Quantitative	Cross-sectional	Primary
Vicuña, 2015	Mixed	Cross-sectional/Interview with key actors	Secondary (CORDAP Report 2013; CASEN 2009 and 2011; the Second National Registry of Homeless People 2012; and CENSUS 1982, 1992, and 2002, Jesuit Service to Migrants of Arica; Ministry of the Interior and Public Security and Investigative Police of Chile).
Ministry of the Interior and Public Security, 2016	Quantitative	Cross-sectional	Secondary (Department of Immigration and Foreign Services (Ministry of the Interior and Public Security); CENSUS 2002).
Troncoso, 2018	Mixed	Cross-sectional/Surveys and semi-structured interviews	Primary
National Institute of Statistics, 2018	Quantitative	Cross-sectional	Secondary (Census 2017)
Jesuit Migrant Service, 2019	Quantitative	Cross-sectional/Survey	Primary
Brondi, 2019	Mixed	Cross-sectional/Interview	Secondary (Under-secretariat of the Interior, PDI, Ministry of Foreign Affairs).
Roessler, 2019	Quantitative	Cross-sectional	Secondary (IOM, INE, DEM, Under-secretariat of the Interior, Migrant Voices, CASEN 2017).
National Institute of Statistics, 2019	Quantitative	Cross-sectional	Secondary (PDI border control database)
Roessler, 2020a	Quantitative	Cross-sectional	Secondary (General Student Information System 2014 to 2019, CASEN 2017, INE, and DEM 2018).
Roessler, 2020b	Quantitative	Cross-sectional	Secondary (CASEN 2017, Migrant Voices Survey 2019, National Camp Registry 2019 (MINVU)).
Vicuña, 2020	Mixed	Cross-sectional/Interview	Secondary (INE, DEM, PDI, Prosecutor’s Office, Under-secretariat of the Interior 2020; Jesuit Service to Migrants of Arica 2020; Testimonials from key actors Tarapacá Region 2020).
Cabieses, 2020	Quantitative	Cross-sectional	Primary
National Institute of Statistics, 2020	Quantitative	Cross-sectional	Secondary (PDI)
Roessler, 2021b	Quantitative	Cross-sectional	Secondary (CASEN 2013, 2015, 2017, and 2020. CENSUS 2017).
Roessler, 2021c	Mixed	Cross-sectional/Discussion group	Secondary (CASEN 2013, 2015, 2017, and 2020, CENSUS 2017 General Student Information System (MINEDUC). Discussion group with workers from the Education and Interculturality Area of ​​the Jesuit Migrant Service).
Jesuit Migrant Service, 2021	Quantitative	Cross-sectional	Primary
Roessler, 2021d	Quantitative	Cross-sectional	Secondary (CENSUS 2017, CASEN 2017, INE, and DEM 2018)
Roessler, 2022	Quantitative	Cross-sectional	Secondary (CASEN 2015, 2017, and 2020)
Zúñiga, 2022	Quantitative	Cross-sectional	Secondary (CASEN 2013, 2015, 2017, and 2020)
Migration and human mobility observatory, 2022	Quantitative	Cross-sectional	Primary
World Bank, 2022	Quantitative	Cross-sectional	Primary
Jesuit Migrants Service, 2024a	Quantitative	Cross-sectional	Secondary (administrative data collected by different government agencies and estimates made by INE and SERMIG, CASEN 2022 and surveys conducted by INE that characterize the migrant population socioeconomically).
Jesuit Migrants Service, 2024b	Mixed	Cross-sectional/Interview	Secondary (administrative data from government agencies such as PDI, National Migration Service, UNHCR, Socio-Legal Assistance Program) Primary (semi-structured interviews)
National Migration Service, 2024a	Quantitative	Cross-sectional	Secondary (administrative acts of applications processed and resolved by the National Migration Service).
National Migrations Service, 2024b	Quantitative	Cross-sectional	Secondary (administrative acts of applications processed by the National Migration Service).

**Table 3:** Description of the bio sociodemographic profile of the population under study.

SCIENTIFIC ARTICLES					
1st authorship (year)	Sample	Countries	Sex	Age ( %)	Education Level ( %)
Parson, 2010	1 woman	Peru	Women	————-	No profession
Rojas, 2011	281 adults; 341 students	Ecuador, Peru, Venezuela	Women, Men	Mean in adult population 29 years ± (15–62); Mean in students 12 ± (6–16)	Adult population: Primary education (14.9 %); Secondary education (64.9 %); Higher education (20.2 %). Student population: Primary education (71 %); Secondary education (29 %).
Cabieses, 2012a	2006:268,873 people of 73,720 homes; 2009: 246,924 people of 74,339 homes.	Peru, Argentina, Ecuador, Bolivia and others.	Woman, Man	Population from Chile: <16 years (23.90 %); 16–65 years (65.42 %); >65 years (10.68 %). Migrant population: <16 years (14.6 %); 16–65 years (76.43 %); >65 years (8.96 %).	Population from Chile: No education (7.62 %); Primary (33.89 %), Secondary (31.37 %); University (15.28 %). Migrant population: No education (3.12 %); Primary (20.05 %), Secondary (30.69 %); University (35.79 %).
Cabieses, 2012b	268,873 people of 73,720 homes	Peru, Argentina, Bolivia, Ecuador, and others	Women, Men	Population from Chile: <16 years (25.27 %); 16–65 years (66.41 %); >65 years (8.32 %). Migrant population: <16 years (13.60 %); 16–65 years (79.08 %); >65 years (7.32 %).	Population from Chile: No education (7.39 %); Primary (34.68 %), Secondary (29.68 %); Technical (14.51 %); University (9.86 %). Migrant population: No education (2.38 %); Primary (18.79 %), Secondary (33.02 %); Technical (16.81 %); University (27.32 %).
Cabieses, 2013	268,873 people of 73,720 homes	Peru, Argentina, Ecuador, Bolivia, and others	Women, Men	< 16 years; 16–65 years; > 65 years	No education; primary, secondary; technical and university.
Cabieses, 2017	1272 cases (pregnant women, children, and adolescents)	Peru, Colombia, Haiti, Bolivia, Dominican Republic, Argentina, and Ecuador.	Women, Men	Pregnant women: Mean migrant women: 27 years Mean women from Chile: 26 years. Children and adolescents Migrant population: <1 year (1.3 %); 1- 6 years (22.8 %); 7–14 years (49.6 %) 15–18 years (26.3 %) Population from Chile: <1 year (4.5 %); 1- 6 years (31.2 %); 7–14 years (40.7 %) 15–18 years (23.7 %)	Pregnant women:———– Children and adolescents: School migrant population (86.1 %) School population from Chile (80.7 %)
Bernales, 2018	256	Peru, Colombia, Ecuador, Bolivia, Dominican Republic, Haiti.	Women, Men	19–69 years	————
Grande, 2019	———-	Peru	Women	15–49 years	Women in Peru 2007: Primary education or less (26.6 %); Secondary education (57.2 %); Higher education (16.2 %). Peruvian women in Chile 2012: Primary education or less (9.2 %); Secondary education (76.6 %); Higher education (14.2 %). Peruvian women in Spain 2007: Primary education or less (8.2 %); Secondary education (58.4 %); Higher education (33.4 %).
Carreño, 2020	14 people: 8 asylum seekers and refugees; 4 public health officials, and 2 from international protection institutions.	Venezuela, Colombia, Peru, Chile	Women, Men	Adults	————–
Caqueo-Urízar, 2021	322 primary students. 312 secondary students.	Colombia	Women, Men	8 to 13 years, mean 10.1 years, SD: 0.9 years 8 to 19 years, mean 14.3 years, SD: 1.8 years	Primary education, Secondary education.
Segovia, 2021	12 Afro-Colombian women	Peru, Bolivia, and Chile	Women	26 to 55 years, mean 37.9 years.	The majority completed secondary education.
Roessler, 2021a	1006 migrants	Venezuela	Men and Women	18 to 29 years (40 %), 30 to 49 years (50 %); 50 or more (10 %).	Secondary education or less (25 %), University education (75 %).
Chen, 2022	95 people of 8 neighborhoods	Haiti	Women, Men	27– 39 years, mean: 33 years.	Incomplete primary education (13 %), complete primary education (44 %), complete secondary education (40 %), complete university education (3 %).
Margarit, 2022a	———-	Venezuela, Peru, Colombia, Haiti, Ecuador, Dominican Republic, China, Bolivia, other country	Men and Women	0 to 19 years (16 %) 20 to 59 years (84 %) 60 years or more (4 %)	Complete higher education in the migrant population (59 %), Complete higher education in the population from Chile (57 %) Primary education in the migrant population (8 %) Completed higher education in the population from Chile (13 %)
Margarit, 2022b	1020 migrants	Colombia, Haiti, Peru, Venezuela, other	Men and Women	18 to 29 years (30.5 %), 30 to 54 years (62.7 %); 55 or more years (6.9 %).	Higher education (43 %)
Barahona, 2022	———-	Statistical Data: Venezuela (23.1 %), Peru (18 %), Haiti (14.3 %), Colombia (11.7 %), Bolivia (8.6 %), and Argentina (3.9 %).	———-	0–14 years (12.65 %), 15–24 years (16.92 %), 25–34 years (42.18 %),35- 44 years (17.41 %), 45 −54 years (6.43 %), 55 years or more (4.41 %)	Technical studies (9.73 %) University studies (61.36 %) Student (23.88 %) Home owner (5.04 %)
Blukacz, 2023	30 migrants	Venezuela (23 %) Peru (17 %) Colombia (17 %) Haiti (10 %) Bolivia (7 %) Ecuador (7 %) Argentina (7 %) Brazil (7 %) Cuba (3 %) Uruguay (3 %)	Men and Women	25–49 years (40 %), 30–35 years (30 %) 36–40 years (13 %) 41–45 years (10 %) >45 años (7 %)	————–
Iribarne-Wiff, 2023	Qualitative: 37 documents. Quantitative:——	———–	———–	———–	————
Baeza-Rivera, 2023	283 migrants	Colombia (13 %) Venezuela (83 %) Haití (4 %)	Men and Women	>18 years	High school complete or incomplete (30 %) University complete or incomplete (62 %) Postgraduate (8 %)
Castillo Lobos, 2023	39 migrants	Venezuela 18 Peru 11 Haiti 9	Women	———–	————
Oyarte, 2023	1841,185 migrants	Peru Argentina Bolivia Ecuador, Venezuela Dominican Republic Colombia Haiti	Men, women	0–18 years, 19–30 years, 31–65 years 66 years or more	————–
OFFICIAL REPORTS					
1st authorship (year)	Sample	Countries of origin	Sex	Age ( %)	Education Level ( %)
Desplanque, 2012	14 refugee cases and 4 social pastoral personnel	Palestine	Men- Women	Adults	Low level of education and illiteracy.
Rojas, 2014	220 migrants	Bolivia, Peru, Colombia	Women, Men	————–	No studies (3.6 %); Incomplete primary education (8.6 %); Complete primary education (9.5 %); Incomplete secondary education (11.8 %); Complete secondary education (39.1 %); Incomplete technical education (7.7 %); Complete technical education (11.4 %); Incomplete university education (5.5 %); Complete university education (2.7 %)
International Migration Organization Chile, 2015	1041 people:	Brazil, Colombia, Ecuador, Argentina, Haiti, Peru.	Men- Women	18–29 years (34.9 %), 30–45 years (50.6 %); 46 years or more (14.5 %). Mean: 34 years.	Primary education (36.9 %); Complete secondary education (24.2 %); Complete or incomplete higher education (13.4 %); No formal studies (1.5 %).
Vicuña, 2015	————-	Bolivia, Colombia, Ecuador, Peru.	Feminine-Masculine	0–14 years (22.7 %); 15–29 years (24.5 %), 30–44 years (18.86 %); 45- 64 years (22.9 %); 65 years or more (11 %).	Professional (4.7 %); Higher level technician (3.7 %); Professional technical secondary education (16.8 %): Technical commercial, industrial, or teacher (1.1 %); Humanistic scientific secondary education (22.1 %); Humanities (old system) (0.5 %); Primary or secondary education (3.2 %); Basic education (38.9 %); Kindergarten/Preschool (1.6 %); Never attended school (7.4 %).
Ministry of the Interior and Public Security, 2016	————-	Peru, Argentina, Bolivia, Colombia, Ecuador, Spain, United States, Brazil, Venezuela, China, and other countries.	Men- Women	0–19 years (21.6 %); 20–35 years (43.3 %), 36–50 years (22.2 %); 51- 65 years (8 %); 66 years or more (4.8 %).	———–
Troncoso, 2018	269 valid cases	Haiti, Venezuela, Colombia.	————-	—————	Incomplete and complete primary education (approx. 8 %); Incomplete and complete secondary education (approx. 52 %); Incomplete and complete technical education (approx. 15 %); Incomplete, complete and postgraduate university education (approx. 27 %)
National Institute of Statistics, 2018	746,465 people	Peru, Colombia, Venezuela, Bolivia, Argentina, Haiti, Ecuador, Spain, Brazil, United States, Dominican Republic, China, Cuba, Mexico, Germany, France; Uruguay, Paraguay, Italy, other country, country not mentioned.	Men- Women	0–14 years, 15–64 years; 65 years or more.	Average education level 25 years or more migrant population: 12.6 men and 12.5 women (average 12.6). Average education level 25 years or more native population: 11.1 men and 10.9 women (average 11) Primary education in migrant (12.8 %) and native (26.1 %) populations; Secondary education in migrant and native populations (44.5 % respectively); Higher education in migrant (42.6 %) and native population (29.1 %)
Jesuit Migrant Service, 2019	1025 people	Venezuela, Peru, Haiti, Colombia, Bolivia, Argentina, Paraguay, other.	Men- Women	18–29 years (30.4 %), 30–54 years (62.5 %); 55 years or more (7.1 %). Mean: 36 years.	No formal studies (0.3 %), incomplete primary education (3.6 %), complete primary education (5 %), incomplete secondary education (15 %), complete secondary education (33 %), incomplete technical education (3.1 %), complete technical education (12.5 %), incomplete higher education (6.3 %), complete higher education (18 %), postgraduate (master/doctorate) (3 %)
Brondi, 2019	————-	Mainly Venezuelan migrants.	Men- Women	Global mean: 39 years. Mean men: 35 years Mean minors: 9 years	————
Roessler, 2019	————-	Bolivia, Colombia, Haiti, Peru, Venezuela.	Men- Women	Migrant population of 20–39 years (58.9 %), total population (31.3 %). Migrant population of 65 years or more (2.8 %), total population (11.8 %).	There is no information on educational level, but there is information on schooling of the migrant population: Preschool Education (4 %); Primary education (4.5 %); Secondary education (4.5 %).
National Institute of Statistics, 2019	Stories of 67 Venezuelans Survey of 110 people	Peru, Argentina, Bolivia, Brazil, United States, Colombia, Venezuela, Spain, France, Germany and rest of countries.	————-	————	————-
Roessler, 2020a	————-	—————–	————–	————	————-
Roessler, 2020b	————-	Peru, Haiti, Bolivia, Venezuela, Colombia, Argentina, and other.	————–	————	————-
Vicuña, 2020	————-	Bolivia, Peru, Haiti, Colombia, Cuba, Syria, other.	Men- Women	Arica and Parinacota Region (RAandT) 20–49 years: 66 % women; 67 % men; 60 years and more: 10 % men; 8 % women Tarapacá Region (RT) 20–49 years: 71 % women; 72 % men; 60 years and more: 4 % men; 3 % women. The percentage of <14 years old is similar.	————-
Cabieses, 2020	1690 Participants	35 countries, the top 4 being: Venezuela, Colombia, Haiti, and Peru.	Men- Women	Mean men: 38.25 years (min 18- max.72.2) Mean women: 38.14 years (min 18-max 87.3) Mean others:35 years.	Men: University education (70.67 %); Secondary education (27.32 %); Primary education (1.82 %); No data (0.18 %) Women: University education (75.83 %); Secondary education (22.06 %); Primary education (1.76 %); No data (0.35 %)
National Institute of Statistics, 2020	————-	Argentina, Peru, Bolivia, Brazil, United States, Colombia, Spain, Venezuela, France, Germany, and rest of the countries.	Men- Women	————–	————-
Roessler, 2021b	————-	Andean countries (Colombia, Peru, Ecuador, and Bolivia), Venezuela, other countries from South America (Argentina, Brazil, Uruguay and Paraguay), Central America (Haiti, Dominican Republic, Cuba, Mexico, among others), Europe, and North America.	Men- Women	< 18 years (26 %), 18–29 years (16 %), 30–59 years (15.2 %); 60 years or more (10.4 %).	Primary education or less (31 %), Secondary education (17 %), Higher education (7 %)
Roessler, 2021c	————-	Neighboring Andean countries, Northern Andean countries, Venezuela, other countries from South America, and Central America.	Men- Women	4–5 years, 6–14 years, 15–18 years, 19–24 years, 26–40 years, 41- 59 years, 60 years or more.	Migrant population over 25 years of age: Primary education or less (17 %), Secondary education (39 %), Higher education (44 %) Native population over 25 years of age: Primary education or less (38 %), Secondary education (36 %), Higher education (26 %)
Jesuit Migrant Service, 2021	1020 people	Venezuela, Peru, Haiti, Colombia, Bolivia, Argentina, and another country.	Men- Women	Mean: 38 years. 18 to 29 years (28.6 %); 30 to 54 years (61.4 %); 55 years or more (10 %).	No formal studies (0.4 %), Incomplete basic education (3.4 %), Complete basic education (5.5 %), Incomplete secondary education (10.9 %), Complete secondary education (36.6 %), Incomplete technical education (6 %), Complete technical education(12.1 %), Incomplete higher education (6.7 %), Complete higher education (15.8 %), Postgraduate (master/doctorate)(2.6 %)
Roessler, 2021d	————-	Peru, Venezuela, Colombia, Haiti, and Bolivia.	Men- Women	15 to 64 years (87 %). 65 years or more (3 %)	Migrant population over 25 years of age: Primary education (12.8 %); Secondary education (44.5 %); Higher education (42.6 %). Native population over 25 years of age: Primary education (26.1 %); Secondary education (44.5 %); Higher education (29.1 %).
Roessler, 2022	————-	Neighboring Andean countries, Northern Andean countries, Venezuela, other countries from South America, Central America, Europe, and North America.	Feminine-Masculine	<18 years, 18–29 years; 30–59 years; 60 years or more.	Primary education or less; Secondary education; Higher education.
Zúñiga, 2022	————-	Neighboring Andean countries (Peru and Bolivia), Northern Andean countries (Colombia and Ecuador), Venezuela, other countries from South America (Argentina, Brazil, Paraguay, and Uruguay), Central America (Haiti, Dominican Republic, Cuba, Mexico, among others); Europe and North America.	Men- Women	15–29 years, 30–59 years; 60 years or more.	Men: Primary education or less Andean countries (29 %), Venezuela (6 %), others South America (21 %), Central America (34 %); Europe and North America (8 %). Secondary education Andean countries (53 %), Venezuela (36 %), others South America (44 %), Central America (50 %); Europe and North America (38 %). Higher education Andean countries (18 %), Venezuela (58 %), others South America (35 %), Central America (16 %); Europe and North America (54 %). Women: Basic education or less Andean countries (27 %), Venezuela (8 %), others South America (21 %), Central America (42 %); Europe and North America (8 %). Secondary education Andean countries (52 %), Venezuela (27 %), others South America (40 %), Central America (44 %); Europe and North America (27 %). Higher education Andean countries (21 %), Venezuela (65 %), others South America (39 %), Central America (15 %); Europe and North America (65 %).
Migration and human mobility observatory, 2022	2001 people	Venezuela, Haiti, Colombia, Andean countries (Peru, Bolivia, others)	Men- Women	18 to 29 years (30.0 %); 30 to 59 years (66.5 %); 60 years or more (3.6 %)	Basic/primary education (3 %); Middle/secondary education (21 %); Higher, university, or technical education (68 %); Postgraduate (8 %).
World Bank, 2022	754,492 people	Venezuela, Peru, Bolivia, Colombia, Haiti, other nationalities.	————	0–15 years (16.5 %); 18–29 years (32 %), 30–39 years (39 %),40- 49 years (17 %), 50 - 59 years (7 %), 60 years or more (4 %). Mean: 35.6 years, Median: 33 years	Primary education or less (16.2 %); Complete secondary education (42.3 %); Complete tertiary education (41.5 %).
Jesuit Migrants Service, 2024a	Different numbers of migrants according to source of information	Venezuela; Peru; Colombia; Haiti; Bolivia; Argentina.	Men, women	———–	———–
Jesuit Migrants Service, 2024b	Different numbers of migrants by source of information 12 interviews with Chilean personnel from private and public institutions.	Bolivia; Colombia; Cuba; Haiti; Peru; Venezuela y otros.	Men, women	<18 years >18 years	———-
National Migrations Service, 2024a	2139,530 permits	Venezuela, Peru, Colombia, Haiti, Bolivia, Cuba, Dominican Republic y others nationalities.	Men, women	17 year or less; 18–29 years; 30–44 years; 45–59 years; 60 years or more.	—————
National Migrations Service, 2024b	89,288 permits	Venezuela (Men:8653; Women: 8362) Colombia (Men: 6189; Women: 7147), Peru (Men: 4599; Women: 4607); Bolivia (Men: 4032; Women: 4436); Ecuador (Men: 1338; Women: 1469); Haiti (Men: 950; Women: 901), Argentina (Men: 886; Women: 708), Other nationalties (Men: 1910; Women: 1922)	Men, women	17 year or less; 18–29 years; 30–44 years; 45–59 years; 60 years or more.	———

**Table 4:** Description of the approach to migration in official articles and reports until 2024.

SCIENTIFIC ARTICLES					
1st authorship (year)	Migration Status	Type of migration	Cultural gap	Acculturation	Health dimension
Parson, 2010	Yes (permanent working visa)	Yes (International)	No	No	No
Rojas, 2011	No	No	No	No	Yes (Mental health and access barriers for assistance)
Cabieses, 2012a	No	No	No	Yes	Yes (disability)
Cabieses, 2012b	No	No	No	No	Yes (access and use of healthcare services)
Cabieses, 2013	No	No	No	No	Yes (disability, health problem, accident, chronic disease, or cancer)
Cabieses, 2017	No	Yes (International)	No	Yes	Yes (health access and use of services)
Bernales, 2018	Yes (irregular)	Yes (International)	Yes	No	Yes (health of the native and migrant population, solution strategies, access and use of healthcare services, impact on the health situation)
Grande, 2019	No	Yes (International)	No	No	No
Carreño, 2020	Yes (asylum/refuge)	Yes (forced/involuntary)	No	Yes	Yes (mental health)
Caqueo-Urízar, 2021	No	Yes (International)	No	Yes	Yes (mental health)
Segovia, 2021	No	Yes (International)	Yes	No	No
Roessler, 2021a	No	Yes (international, humanitarian)	No	No	Yes (health insurance, access to information)
Chen, 2022	No	Yes (International)	No	No	Yes (mental health)
Margarit, 2022a	No	No	Yes (language)	No	No
Margarit, 2022b	Yes (with/without permanent permission)	No	Yes (language)	No	No
Barahona, 2022	Yes (permanent visa)	No	No	No	No
Blukacz, 2023	Yes (visa in process, temporary visa in force, definitive visa and irregular status)	Yes (International)	Yes (language)	No	Yes (access barriers for assistance in COVID19 pandemic)
Iribarne-Wiff, 2023	No	No	No	No	Yes (health insurance, hospital discharges, not having received medical attention)
Baeza-Rivera, 2023	No	Yes (International)	No	No	Yes, satisfaction with health personnel (competence and skills, attitude, empathy, and interest in the empathy, and interest in wellness and time of dedication, communication skills, experience and proximity), and aspects of improvement (critical and constructive and proximity), and aspects of improvement (critical and constructive attitude).
Castillo Lobos, 2023	———	Yes (international)	Yes (language, parenting in a migrant situation)	Yes (definition and four different orientations of acculturation)	Mental health, physical health, and health services
Oyarte, 2023	———–	Yes (international)	———–	————-	Access and use of health services
OFFICIAL REPORTS					
1st authorship (year)	Migration Status	Type of migration	Cultural gap	Acculturation	Health dimension
Desplanque, 2012	No	Yes (humanitarian)	Yes (language)	No	Yes (reproductive and gynecological health)
Rojas, 2014	Yes (temporary working visa, permanent working visa, tourist, irregular, asylum/refuge, among others)	Yes (international, irregular, regular, humanitarian)	No	No	No
International Migration Organization Chile, 2015	Yes (temporary, permanent, tourist, subject to a work contract, student)	Yes (international, humanitarian)	Yes (language)	No	No
Vicuña, 2015	Yes (Tacna-Arica agreement)	Yes (International)	Yes (language)	No	Yes (morbidity, preventive control, hospitalization/surgical intervention, health insurance, care needs, user satisfaction)
Ministry of the Interior and Public Security, 2016	Yes (temporary, subject to contract, students)	Yes (international, humanitarian)	Yes (language)	No	No
Troncoso, 2018	Yes (temporary, permanent, irregular)	No	Yes (language)	No	No
National Institute of Statistics, 2018	No	No	Yes (language)	No	No
Jesuit Migrant Service, 2019	Yes (permanent, temporary, subject to contract, expired pending renewal, expired without processing, irregular, tourist, student, dependent, other)	Yes (International)	Yes (language)	No	No
Brondi, 2019	Yes (regular, irregular, in process)	Yes (international, humanitarian)	No	No	No
Roessler, 2019	Yes (temporary, permanent, consular)	Yes (international, humanitarian)	Yes (language)	No	No
National Institute of Statistics, 2019	Yes (permanent, tourist)	Yes (International)	Yes (language)	No	No
Roessler, 2020a	No	No	No	No	No
Roessler, 2020b	Yes (temporary, permanent, tourist, irregular)	No	Yes (language)	No	No
Vicuña, 2020	Yes (asylum seekers, irregular)	Yes (international, irregular, forced/involuntary, humanitarian)	Yes (language)	No	No
Cabieses, 2020	No	No	Yes (language)	No	Yes (health insurance)
National Institute of Statistics, 2020	Yes (permanent, tourist)	Yes (International)	Yes (language)	No	No
Roessler, 2021b	No	No	Yes (language)	No	No
Roessler, 2021c	No	No	Yes (language)	No	No
Jesuit Migrant Service, 2021	No	Yes (International)	Yes (language)	No	No
Roessler, 2021d	No	No	Yes (language)	No	No
Roessler, 2022	No	No	Yes (language)	No	Yes (access to healthcare and gaps)
Zúñiga, 2022	No	No	No	No	No
Migration and human mobility observatory, 2022	No	Yes (international, humanitarian)	Yes (language)	No	No
World Bank, 2022	No	Yes (International)	Yes (language)	No	Yes (differences in access to the healthcare system according to occupational category)
Jesuit Migrants Service, 2024a	Yes (permanent, temporary, asylum seekers, tourist, student, subject to contract, others)	Yes (international, forced/involuntary, humanitarian)	————–	—————-	Yes (access and use of basic health care services, types of health insurance, access to and use of mental health care services)
Jesuit Migrants Service, 2024b	Yes (regular, irregular, in process, asylum seekers, refugees)	Yes (international, irregular, forced/involuntary, humanitarian)	————–	No intercultural approach to the detection of human trafficking victims.	Lack of training of public health officials, Right to care for pregnant women.
National Migrations Service, 2024a	Yes (applications accepted and granted for temporary and definitive residence)	Yes (international, forced/involuntary, humanitarian)	————-	—————	——————
National Migrations Service, 2024b	Yes (applications granted)	Yes (international, humanitarian)	————-	—————	Yes (right to health care for pregnant women)

## 3 Results

### 3.1 Research landscape: characteristics of articles and officially reports included

**Fig. 1:**
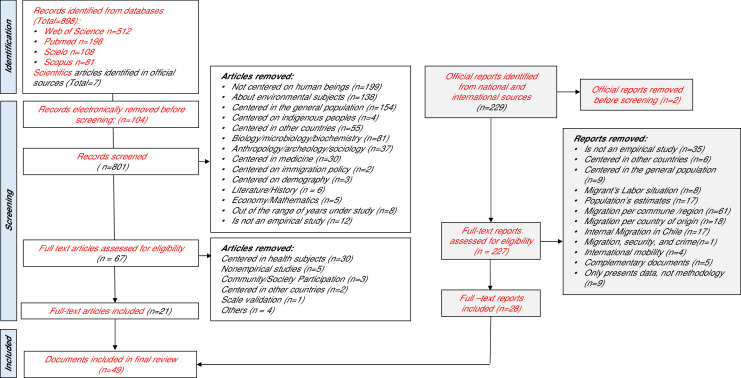
PRISMA Flowchart of included articles and reports.

By searching in the scientific databases, initially 898 articles were identified. After the exclusion process (see Fig. 1 PRISMA Flow Chart), 21 articles were added. As for the official reports, 229 reports were identified, and after the exclusion analysis, 28 reports were added.

Regarding the general characteristics of the articles, scientific publications were concentrated from 2010 to 2023, with 57 % of the publications (*n* = 12) between 2020 and 2023. In contrast, the first authors were mainly from Chile (Baeza-Rivera, 2023; Bernales, 2018; Blukacz, 2023; Cabieses, 2012a, 2012b; Cabieses, 2013; Cabieses, 2017; Caqueo-Urízar, 2021; Carreño, 2020; Castillo-Lobos, 2023; Iribarne-Wiff, 2024; Margarit, 2022a; Margarit, 2022b; Oyarte, 2023; Roessler, 2021a; Rojas, 2011; Segovia, 2021;) with notable contributions from international authors, including authors from the United States (Chen, 2022; Parson, 2010) and Spain (Barahona, 2022; Grande, 2019). Furthermore, 76 % (*n* = 16) of the journals in which articles were published focused on healthcare subjects (Baeza-Rivera, 2023; Bernales, 2018; Blukacz, 2023; Cabieses, 2012a, 2012b; Cabieses, 2013; Cabieses, 2017; Castillo-Lobos, 2023; Chen, 2022; Caqueo-Urízar, 2021; Carreño, 2020; Iribarne-Wiff, 2024; Oyarte, 2023; Roessler, 2021a; Rojas, 2011; Segovia, 2021). Only one article was published in a migration journal (Grande, 2019). Regarding the object of study, the articles mainly focused on measuring mental health (MH) (Caqueo-Urízar, 2021; Carreño, 2020; Chen, 2022; Rojas, 2011), access/use of healthcare services (Blukacz, 2023; Cabieses, 2012a; Oyarte, 2023; Rojas, 2011), and describing the profile or the socioeconomic characteristics of the migrant population (Barahona, 2022; Bernales, 2018; Cabieses, 2012b; Roessler, 2021a; Segovia, 2021) (see Table 1).

### 3.2 Regarding the general characteristics of the reports

The scientific publications were concentrated from 2012 to 2024, with 57 % of the publications (*n* = 16) between 2020 and 2024. The institutions responsible for publishing the reports included the Jesuit Migrant Service (Brondi, 2019; Cabieses, 2020; Jesuit Migrant Service, 2019, 2021; Jesuit Migrants Service, 2024a, 2024b; Migration and human movility observatory, 2022; Roessler, 2020a, 2020b, 2021b; Roessler, 2021c, 2021d, 2022; Rojas, 2014; Troncoso, 2018; Vicuña, 2020; Zúñiga, 2022), accounting for 57 % (*n* = 16), the academic community comprising national universities (Cabieses, 2020; Desplanque, 2012; International Organization for Migration, 2015; Roessler, 2020a, 2020b, 2021d; World Bank, 2022), representing 18 %, foundations (Roessler, 2019, 2020a, 2020b; Troncoso, 2018; Vicuña, 2015) at 14 %, as well as international organizations such as the World Bank (World Bank, 2022), International Organization for Migration (International Organization for Migration, 2015; Rojas, 2014) and United Nations High Commissioner for Refugees (UNHCR) (International Organization for Migration, 2015) . Regarding the object of study of the reports, 54 % (*n* = 15) focused on describing the characteristics of the migrant population, 18 % (*n* = 5) on access to services (housing, education, or healthcare), and 14 % on the labor situation of the migrant population (*n* = 4) to recognize the existing gaps. (See Table 1).

### 3.3 As for the methodological approach of the scientific articles

50 % (*n* = 14) adopted a quantitative approach. All of the studies were cross-sectional observations (Baeza-Rivera, 2023; Barahona, 2022; Cabieses, 2012a, 2012b, 2013; Cabieses, 2017; Caqueo-Urízar, 2021; Chen, 2022; Grande, 2019; Iribarne-Wiff et al., 2024; Margarit, 2022a, 2022b; Oyarte, 2023; Roessler, 2021a; Rojas, 2011). From these, 32 % (*n* = 9) used secondary data for the analysis (Barahona, 2022; Cabieses, 2012b, 2013, 2017; Grande, 2019; Iribarne-Wiff et al., 2024; Margarit, 2022a, 2022b; Oyarte, 2023) (see Table 2).

### 3.4 As for the methodological approach of the official reports

96 % (*n* = 27) adopted a quantitative approach. All of the studies were cross-sectional observations (Brondi, 2019; Cabieses, 2020; International Organization for Migration, 2015; Jesuit Migrant Service, 2019, 2021; Jesuit Migrants Service, 2024a, 2024b; Migration and human mobility observatory, 2022; Ministry of the Interior and Public Security, 2016; National Institute of Statistics, 2018, 2019, 2020; National Migration Service, 2024a, 2024b; Roessler, 2019, 2020a, 2020b; Roessler, 2021b, 2021c, 2021d; Roessler, 2022; Rojas, 2014; Troncoso, 2018; Vicuña, 2015, 2020; World Bank, 2022; Zúñiga, 2022). From these, 22 % (*n* = 6) of these articles added a qualitative stage to the described study (Brondi, 2019; Jesuit Migrants Service, 2024b; Roessler, 2021c; Troncoso, 2018; Vicuña, 2020, 2015). Of the reported total, 70 % (*n* = 19) used secondary data for the analysis (see Table 2).

### 3.5 Analyzing the characteristics of the population under study

It was highlighted that, within the articles, the sample size ranged from an ethnographic study of 1 person (Parson, 2010) to 1841,185 migrants (Oyarte, 2023) persons. In the case of the reports, the sample ranged between 18 (Desplanque, 2012) and 2139,530 (National Migrations Service, 2024a) persons. When identifying the countries of origin of the migrant population, in both publications, the predominant countries were Peru (Articles: 76 % (*n* = 16) (Barahona, 2022; Bernales, 2018; Blukacz, 2023; Cabieses, 2012a, 2012b, 2013; Cabieses, 2017; Carreño, 2020; Castillo Lobos, 2023; Grande, 2019; Margarit, 2022a, 2022b; Oyarte, 2023; Parson, 2010; Rojas, 2011; Segovia, 2021); Reports: 86 % (*n* = 24) (Cabieses, 2020; International Organization for Migration, 2015; Jesuit Migrant Service, 2019; Jesuit Migrant Service, 2021; Jesuit Migrant Service, 2024a; Jesuit Migrant Service, 2024b; Migration and human movility observatory, 2022; Ministry of the interior and public security, 2016; National Institute of Statistics, 2018; National Institute of Statistics, 2019, 2020; National Migrations Service, 2024a, 2024b; Roessler, 2019, 2020b, 2021b;Roessler, 2021c, 2021d, 2022; Rojas, 2014; Vicuña, 2015, 2020; World Bank, 2022; Zúñiga, 2022), Colombia (Articles: 44 % (*n* = 7) (Barahona, 2022; Bernales, 2018; Cabieses, 2017; Caqueo-Urízar, 2021; Carreño, 2020; Margarit, 2022a, 2022b) Reports: 83 % (*n* = 20) (Cabieses, 2020; International Organization for Migration, 2015; Jesuit Migrant Service, 2019, 2021; Migration and human mobility observatory, 2022; Ministry of the interior and public security, 2016; National Institute of Statistics, 2018; National Institute of Statistics, 2019, 2020; Roessler, 2019, 2020b, 2021b; Roessler, 2021c, 2021d; Rojas, 2014; Troncoso, 2018; Vicuña, 2015, 2020; World Bank, 2022; Zúñiga, 2022), and Bolivia (Articles: 50 % (*n* = 8) (Barahona, 2022; Bernales, 2018; Cabieses, 2012a, 2012b, 2013; Cabieses, 2017; Segovia, 2021; Margarit, 2022a); Reports: 75 % (*n* = 18) (Jesuit Migrant Service, 2019, 2021; Migration and human mobility observatory, 2022; Ministry of the interior and public security, 2016; National Institute of Statistics, 2018; National Institute of Statistics, 2019, 2020; Roessler, 2019, 2020b, 2021b; Roessler, 2021c; Roessler, 2021d; Roessler, 2022; Rojas, 2014; Vicuña, 2015, 2020; World Bank, 2022; Zúñiga, 2022). In the articles, Ecuador was also highlighted, with 44 % (*n* = 7) (Bernales, 2018; Cabieses, 2012a, 2012 b; Cabieses, 2013; Cabieses, 2017; Margarit, 2022a; Rojas, 2011). In the reports, Venezuela was highlighted, with 75 % (*n* = 18) (Brondi, 2019; Cabieses, 2020; Jesuit Migrant Service, 2019, 2021; Migration and human mobility observatory, 2022; Ministry of the interior and public security, 2016; National Institute of Statistics, 2018; National Institute of Statistics, 2019, 2020; Roessler, 2019, 2020b, 2021b; Roessler, 2021c, 2021d, 2022; Troncoso, 2018; World Bank, 2022; Zúñiga, 2022). The rest of the countries under study are presented in Table 3. Only one article mentioned a country other than Chile as a destination. The study by Chen and et al. described Chile as a temporary destination country for the population from Haiti and for whom the destination country for the future will be the United States (Chen, 2022).

Furthermore, 91 % (*n* = 19) of the articles included the sex of the population in the study (Baeza-Rivera, 2023; Bernales, 2018; Blukacz, 2023; Cabieses, 2012a, 2012b, 2013; Cabieses, 2017; Caqueo-Urízar, 2021; Carreño, 2020; Castillo Lobos, 2023; Chen, 2022; Grande, 2019; Margarit, 2022a, 2022b; Oyarte, 2023; Parson, 2010; Rojas, 2011; Roessler, 2021a; Segovia, 2021). All the articles included women, and 71 % (*n* = 15) included men (Baeza-Rivera, 2023; Bernales, 2018; Blukacz, 2023; Cabieses, 2012a, 2012b, 2013; Cabieses, 2017; Caqueo-Urízar, 2021; Carreño, 2020; Chen, 2022; Margarit, 2022a, 2022b; Oyarte, 2023; Roessler, 2021a; Rojas, 2011). Moreover, 82 % (*n* = 23) of the reports included the sex of the population in the study (Brondi, 2019; Cabieses, 2020; Desplanque, 2012; International Organization for Migration, 2015; Jesuit Migrant Service, 2019, 2021; Jesuit Migrants Service, 2024a, 2024b; Migration and human mobility observatory, 2022; Ministry of the interior and public security, 2016; National Institute of Statistics, 2018; National Institute of Statistics, 2020; National Migrations Service, 2024a; Roessler, 2019, 2021b, 2021c; Roessler, 2021d, 2022; Rojas, 2014; Vicuña, 2015, 2020; Zúñiga, 2022), including both women and men (See Table 3). Regarding the age of the population under study, 86 % (*n* = 18) of the articles specified the age, ranging from <1 year (Cabieses, 2017) to 69 years (Bernales, 2018), and 75 % (*n* = 21) of the reports specified this variable (Brondi, 2019; Cabieses, 2020; Desplanque, 2012; Jesuit Migrant Service, 2019, 2021; Jesuit Migrants Service, 2024a, 2024b; Migration and human mobility observatory, 2022; Ministry of the interior and public security, 2016; National Institute of Statistics, 2018; National Migrations Service, 2024a; Roessler, 2019, 2021b, 2021c; Roessler, 2021d, 2022; Rojas, 2014; Vicuña, 2015, 2020; World Bank, 2022; Zúñiga, 2022), ranging from 0 to 14 years (Troncoso, 2018; Vicuña, 2015) to 66 or more (Ministry of the interior and public security, 2016; National Migrations Service, 2024a, 2024b) (See Table 3).

When analyzing the education level of the population under study, 71 % (*n* = 15) of the articles specified the level (Baeza-Rivera, 2023; Barahona, 2022; Cabieses, 2012a, 2012b, 2013; Cabieses, 2017; Caqueo-Urízar, 2021; Chen, 2022; Grande, 2019; Margarit, 2022a, 2022b; Parson, 2010; Roessler, 2021a; Rojas, 2011; Segovia, 2021) but 87 % (*n* = 13) only specified the category of the education (primary, secondary, technical, or higher education) (Baeza-Rivera, 2023; Barahona, 2022; Cabieses, 2012a, 2012b, 2013; Caqueo-Urízar, 2021; Chen, 2022; Margarit, 2022a, 2022b; Roessler, 2021a; Rojas, 2011; Segovia, 2021). However, 69 % of these articles (*n* = 9) indicated in detail the percentage of these categories (Baeza-Rivera, 2023; Barahona, 2022; Cabieses, 2012a, 2012b; Chen, 2022; Margarit, 2022a, 2022b; Roessler, 2021a; Rojas, 2011), and only 23 % (*n* = 3) indicated a comparison between the population from Chile and the migrant population (Cabieses, 2012a, 2012b; Margarit, 2022a). Two articles highlighted that the migrant population exhibited fewer figures in primary education and greater figures in higher education than the population from Chile (Cabieses, 2012a, 2012b). (See Table 3).

In the case of official reports, 61 % (*n* = 17) recorded the level of education of the population under study (Cabieses, 2020; Desplanque, 2012; International Organization for Migration, 2015; Jesuit migrant Service, 2019, 2021; Migration and human mobility observatory, 2022; National institute of Statistics, 2018; Roessler, 2019; Roessler, 2021b, 2021c, 2021d; Roessler, 2022; Rojas, 2014; Troncoso, 2018; Vicuña, 2015; World Bank, 2022; Zúñiga, 2022. Of these, 94 % (*n* = 16) indicated it as categories (Cabieses, 2020; International Organization for Migration, 2015; Jesuit migrant Service, 2019, 2021; Migration and human mobility observatory, 2022; National institute of s Statistics, 2018; Roessler, 2019, 2021b, 2021c; Roessler, 2021d, 2022; Rojas, 2014; Troncoso, 2018; Vicuña, 2015; World Bank, 2022; Zúñiga, 2022), and 94 % (*n* = 15) indicated it in percentage by category (Cabieses, 2020; International Organization for Migration, 2015; Jesuit migrant Service, 2019, 2021; Migration and human mobility observatory, 2022; National institute of Statistics, 2018; Roessler, 2019, 2021b, 2021c; Roessler, 2021d; Rojas, 2014; Troncoso, 2018; Vicuña, 2015; World Bank, 2022; Zúñiga, 2022). Only 13 % (*n* = 2) indicated a comparison between the population from Chile and the migrant population (Jesuit migrant Service, 2019; National institute of Statistics, 2018). The reports, as well as the articles, highlighted that the migrant population exhibited fewer figures in primary education and greater figures in higher education than the population from Chile. (See Table 3).

As for the occupation of the migrant population, only 43 % of the articles (*n* = 9) included it. In general, it was used as a synonym of the socioeconomic level (SEL). The occupation’s description in the publications used heterogeneous classifications ranging from specific groups such as students (Caqueo-Urízar, 2021; Ministry of the interior and public security, 2016), comparison between active workers versus laid-off workers (Cabieses, 2012b, 2013), and classifications based on the occupation sector such as commerce, domestic service, informal labor and street commerce (Margarit et al., 2022a; Parson, 2010; Castillo-Lobos, 2023). Only 25 % (*n* = 4) used wider classifications that corresponded to international scales (Barahona, 2022; Cabieses et al., 2012a; Rojas, 2014; Segovia, 2021).

From 28 official reports, the occupation variable was present in only 39 % of the reports (*n* = 11), reaching a higher descriptive level than in the articles. The occupation description of the migrant population was presented in various ways: one report only differentiated formal and informal employment (Roessler, 2022); another one compared the migrant population and the native population according to economic sectors (primary, secondary, and tertiary sector) or service sector. In 2017 (National Institute of Statistics, 2018) 87.8 % of the migrant population belonged to the service sector. Another two reports compared the occupation according to sector, distinguishing between students, public and private sector employees, and domestic service (Ministry of the Interior and Public Security, 2016; Zúñiga, 2022). Two more reports compared the evolution of occupation in the migrant population between 2019 and 2021, showing a decrease in occupation quality during the COVID-19 pandemic. This represented a fall from 36.1 % to 22.9 % in the service sector and from 15 % to 3.6 % of scientists and scholars. Meanwhile, during the same period, the occupation of nonqualified jobs (Jesuit Migrant Service, 2019, 2021) increased from 17.8 % to 29.5 %. Finally, 16.6 % (*n* = 4) of the reports offered a detailed perspective of the occupation of specific groups of migrants, such as cross-border migration in Arica and Parinacota, where 50 % of the population works as farm and agricultural workers (Vicuña, 2015), and a group of Venezuelan migrants separated by occupational sectors and by sex (Roessler, 2021d). Another two reports indicated the occupation of the refugee migrant population (Desplanque, 2012; International Organization for Migration, 2015). A report describes employment rates from 72.2 % to 62.2 % between 2018 and 2023, informal employment rate from 2018 (29.1 %) to 2022 (34.1 %), underemployment by skills for the year 2022 among women (19.5 %) and men (15.7 %), and finally, it describes the insertion in the labor force by economic sector between the years 2018 and 2022 (Jesuit Migrants Service, 2024a).

Different characteristics can be observed, both in the articles and in the reports, when addressing different identities of the population under study, such as disability (Cabieses, 2012a), asylum and/or refuge (Brondi, 2019; Carreño, 2020; Desplanque, 2012; Jesuit Migrants Service, 2024a, 2024b; Migration and human mobility observatory, 2022; Ministry of the Interior and Public Security, 2016; National Migrations Service, 2024a; Vicuña, 2020), African descent (Blukacz, 2023; Jesuit Migrants Service, 2024b; Segovia, 2021), and indigenous origin (Jesuit Migrants Service, 2024b; Parson, 2010; Vicuña, 2015).

Recognizing the native language of the migrant population was primarily done by identifying the country of origin. Thus, in 81 % (*n* = 17) (Baeza-Rivera, 2023; Barahona, 2022; Bernales, 2018; Blukacz, 2023; Cabieses, 2012b, 2017; Caqueo-Urízar, 2021; Carreño, 2020; Castillo Lobos, 2023; Chen, 2022; Grande, 2019; Margarit, 2022a, 2022b; Oyarte, 2023; Parson, 2010; Roessler, 2021a; Segovia, 2021) of the articles, the native language was identified, from which 94 % (*n* = 16) (Baeza-Rivera, 2023; Barahona, 2022; Bernales, 2018; Blukacz, 2023; Cabieses, 2012b, 2017; Caqueo-Urízar, 2021; Carreño, 2020; Castillo Lobos, 2023; Grande, 2019; Margarit, 2022a, 2022b; Oyarte, 2023; Parson, 2010; Roessler, 2021a; Segovia, 2021) were in Spanish, 53 % (*n* = 9) in Creole (Baeza-Rivera, 2023; Bernales, 2018; Blukacz, 2023; Cabieses, 2017; Castillo Lobos, 2023; Chen, 2022; Margarit, 2022a, 2022b; Oyarte, 2023), and 7 % (*n* = 1) in Chinese (Ministry of the Interior and Public Security, 2016). In 96 % of the reports (*n* = 27), the native language was identified (Brondi, 2019; Cabieses, 2020; Desplanque, 2012; International Organization for Migration, 2015; Jesuit Migrant Service, 2019, 2021; Jesuit Migrants Service, 2024a, 2024b; Migration and human mobility observatory, 2022; Ministry of the Interior and Public Security, 2016; National Institute of Statistics, 2018; National Institute of Statistics, 2019, 2020; National Migrations Service, 2024a, 2024b; Roessler, 2019, 2020b, 2021b; Roessler, 2021c, 2021d, 2022; Rojas, 2014; Troncoso, 2018; Vicuña, 2015, 2020; World Bank, 2022; Zúñiga, 2022). The majority were in Spanish (96 %; *n* = 26) (Brondi, 2019; Cabieses, 2020; International Organization for Migration, 2015; Jesuit Migrant Service, 2019, 2021; Jesuit Migrants Service, 2024a, 2024b; Migration and human mobility observatory, 2022; Ministry of the Interior and Public Security, 2016; National Institute of Statistics, 2018; National Institute of Statistics, 2019, 2020; National Migrations Service, 2024a, 2024b; Roessler, 2019, 2020b, 2021b; Roessler, 2021c, 2021d, 2022; Rojas, 2014; Troncoso, 2018; Vicuña, 2015, 2020; World Bank, 2022; Zúñiga, 2022), followed by those in Creole (74 %; *n* = 20) (Cabieses, 2020; International Organization for Migration, 2015; Jesuit Migrant Service, 2019, 2021; Jesuit Migrants Service, 2024a, 2024b; Migration and human mobility observatory, 2022; National Institute of Statistics, 2019; National Migrations Service, 2024a, 2024b; Roessler, 2019, 2020b, 2021b; Roessler, 2021c, 2021d, 2022; Troncoso, 2018; Vicuña, 2020; World Bank, 2022; Zúñiga, 2022). The rest of the native languages identified were English (International Organization for Migration, 2015; Jesuit Migrant Service, 2019, 2021; Roessler, 2021b, 2022; Ministry of the Interior and Public Security, 2016; National Institute of Statistics, 2018; National Institute of Statistics, 2019, 2020), French (International Organization for Migration, 2015; Jesuit Migrant Service, 2019, 2021; National Institute of Statistics, 2019, 2020), Chinese (Ministry of the Interior and Public Security, 2016), Arabic (Desplanque, 2012; Vicuña, 2020), German (National Institute of Statistics, 2018; National Institute of Statistics, 2019, 2020), and Italian (National Institute of Statistics, 2018).

### 3.6 Characteristics of migration in Chile

As for the migration approach, 29 % (*n* = 6) (Barahona, 2022; Bernales, 2018; Blukacz, 2023; Carreño, 2020; Margarit, 2022b; Parson, 2010) of the articles and 57 % (*n* = 16) (Brondi, 2019; International Organization for Migration, 2015; Jesuit Migrant Service, 2019; Jesuit Migrants Service, 2024a, 2024b; Ministry of the Interior and Public Security, 2016; National Institute of Statistics, 2019, 2020; National Migrations Service, 2024a, 2024b; Roessler, 2019, 2020b; Rojas, 2014; Troncoso, 2018; Vicuña, 2015, 2020) of the reports exhibited characteristics of the migration situation of the population under study. It was concentrated in the status related to staying in the national territory. Furthermore, 62 % (*n* = 13) (Baeza-Rivera, 2023; Bernales, 2018; Blukacz, 2023; Cabieses, 2017; Caqueo-Urízar, 2021; Carreño, 2020; Castillo Lobos, 2023; Chen, 2022; Grande, 2019; Oyarte, 2023; Parson, 2010; Roessler, 2021a; Segovia, 2021) of the articles and 64 % (*n* = 18) (Brondi, 2019; Desplanque, 2012; International Organization for Migration, 2015; Jesuit Migrant Service, 2019, 2021; Jesuit Migrants Service, 2024a, 2024b; Migration and human mobility observatory, 2022; Ministry of the Interior and Public Security, 2016; National Institute of Statistics, 2019, 2020; National Migrations Service, 2024a, 2024b; Roessler, 2019; Rojas, 2014; Vicuña, 2015, 2020; World Bank, 2022) of the reports indicated the type of migration. The majority focused on the description of international migration (*n* = 29) (Baeza-Rivera, 2023; Bernales, 2018; Blukacz, 2023; Brondi, 2019; Cabieses, 2017; Caqueo-Urízar, 2021; Castillo Lobos, 2023; Chen, 2022; Grande, 2019; International Organization for Migration, 2015; Jesuit Migrant Service, 2019, 2021; Jesuit Migrants Service, 2024a, 2024b; Migration and human mobility observatory, 2022; Ministry of the Interior and Public Security, 2016; National Institute of Statistics, 2019, 2020; National Migrations Service, 2024a, 2024b; Oyarte, 2023; Parson, 2010; Roessler, 2019, 2021a; Rojas, 2014; Segovia, 2021; Vicuña, 2015, 2020; World Bank, 2022), followed by humanitarian migration (*n* = 14) (Brondi, 2019; Carreño, 2020; Desplanque, 2012; International Organization for Migration, 2015; Jesuit Migrants Service, 2024a, 2024b; Migration and human mobility observatory, 2022; Ministry of the Interior and Public Security, 2016; National Migrations Service, 2024a, 2024b; Roessler, 2019, 2021a; Rojas, 2014; Vicuña, 2020). The main cultural gap identified in the articles and official reports was the language, representing 19 % (*n* = 4) (Blukacz, 2023; Castillo Lobos, 2023; Margarit, 2022a, 2022b) and 71 % (*n* = 20) (Cabieses, 2020; Desplanque, 2012; International Organization for Migration, 2015; Jesuit Migrant Service, 2019, 2021; Migration and human mobility observatory, 2022; Ministry of the Interior and Public Security, 2016; National Institute of Statistics, 2018, 2019, ; 2020; Roessler, 2019, 2020b, 2021b; Roessler, 2021c, 2021d, 2022; Troncoso, 2018; Vicuña, 2015, 2020; World Bank, 2022), respectively. Moreover, 24 % (*n* = 5) of the articles (Cabieses, 2012a, 2017; Caqueo-Urízar, 2021; Carreño, 2020; Castillo Lobos, 2023) mentioned the acculturation process, and none of the official reports mentioned this. (See Table 3).

When investigating the causes of migration, 25 % (*n* = 4) of the articles mentioned them, highlighting the search for dreams and a change of life (Chen, 2022), political instability (Carreño, 2020), and lack of economic opportunities (Barahona, 2022; Parson, 2010). As for the official reports, 21 % (*n* = 4) stated as causes the lack of labor and economic opportunities (Brondi, 2019; Jesuit Migrant Service, 2019, 2021; World Bank, 2022; Jesuit Migrant Service, 2024b), political instability, and armed conflicts (Desplanque, 2012; Jesuit Migrant Service, 2024b), discrimination based on sexual identity and orientation, access to children education, fear of attack or persecution (direct threat), asylum seeker, access to health care, insecurity or generalized violence, family reunification, and access to food. (Jesuit Migrant Service, 2024b). In the same vein, but related to the attracting factors of migration toward Chile, 14 % (*n* = 3) of the articles mentioned this (Parson, 2010; Segovia, 2021; Castillo Lobos, 2023), highlighting the labor and economic opportunities of the country. In addition, 21 % (*n* = 5) (Brondi, 2019; International Organization for Migration, 2015; Jesuit Migrant Service, 2019, 2021; World Bank, 2022) of the reports mentioned the attracting factors, similar to the ones previously mentioned but highlighting the employment possibilities (Brondi, 2019; International Organization for Migration, 2015), which decreased between 2019 and 2021 (2019:31.8 %; 2021:27.8 %) (Jesuit Migrant Service, 2019, 2021), as well as highlighting having an acquaintance/family member in Chile as an attracting factor, which increased in the same years (2019:31.5 %; 2021:48.1 %) (Jesuit Migrant Service, 2019, 2021). The reports also indicated the differences between countries for the population of Bolivia, Colombia, Haiti, and Venezuela. The most important attracting factor was Chile’s economic stability (World Bank, 2022), family reunification for Peru and other countries, especially for women (World Bank, 2022; Jesuit Migrants Service, 2024b), and the search for quality of life for Haiti (World Bank, 2022).

In addition, 71 % (*n* = 15) of the scientific articles and 29 % (*n* = 8) of the reports included some health dimension in the data analysis. In the first group, the health dimension focused on the access and use of healthcare services (Baeza-Rivera, 2023; Bernales, 2018; Blukacz, 2023; Cabieses, 2012b, 2013; Castillo Lobos, 2023; Iribarne-Wiff, 2023; Oyarte, 2023; Roessler, 2021a; Rojas, 2011), followed by identifying the determinant factors of the population’s mental health (Caqueo-Urízar, 2021; Chen, 2022; Carreño, 2020; Rojas, 2011). In the second group, these dimensions varied, including access to healthcare assistance (Jesuit Migrants Service, 2024a, 2024b; Roessler, 2022; World Bank, 2022), health insurance (Cabieses, 2020; Jesuit Migrants Service, 2024a), several health indicators in the migrant population (Vicuña, 2015), and cultural barriers to access reproductive and gynecological health (Desplanque, 2012). (See Table 4).

Regarding the level of knowledge that the migrant population in Chile has related to accessing healthcare assistance, only 9 (43 %) articles and 4 (14 %) reports identified it. Furthermore, 80 % (*n* = 4) of the articles recognized the lack of knowledge in this area (Cabieses, 2017; Carreño, 2020; Roessler, 2021a; Rojas, 2011), resulting in late access to prenatal care (Cabieses, 2017) in some cases, and the rest of the people were dissatisfied with the assistance they received (Bernales, 2018). There are also reports about the identification of drivers and hindering factors of health care access (Blukacz, 2023); reducing the gap in health care access (Iribarne-Wiff, 2023), high client satisfaction with health care (Baeza-Rivera, 2023) and a description of the difference in Access to health insurance of recent and settled migrant population (Oyarte, 2023). One of the reports conducted in 2020 focused on identifying the lack of knowledge to access mechanisms when showing COVID-19 symptoms. It highlighted that people who had been living in Chile for at least 6 months exhibited the greatest numbers in terms of lack of knowledge of where to go (48.2 %) (Cabieses, 2020). People who had been living in Chile for 6 to 10 years (26.9 %) (Cabieses, 2020) exhibited the lowest numbers. The CASEN and migration report (Roessler, 2022) differentiated between access to healthcare and the absence of access, gross or total (total percentage of people who while having health problems did not access medical attention or went to a consult), and noneffective access (percentage of people who while having health problems did not access medical attention or go to a consult due to barriers that were out of their control) (Roessler, 2022). In this sense, lower gross access was observed between 2015 and 2020 in the migrant population compared with the population from Chile, even though the differences were shorter. In 2015, 11.8 % of foreigners who had health problems did not go to a consult. In 2020, the number decreased to 8.6 %. For the population of Chile, this indicator was more stable between 6.1 % and 7.5 %. This result suggests a decrease in the difference between both populations of 5.7 points in 2015 and 1.1 points in 2020. The figures for noneffective access were similar for migrants and for people from Chile. In 2015, the figures were 1 % and 2 %, respectively. The figures increased to 4 % and 5 %, respectively, due to the pandemic barriers (Roessler, 2022). Finally, one report describes the general health access characteristics (medical checkups for children 5 to 9 years old and adolescents) and mental health, as well as hospital discharges and the percentage of households without health care access by region (Jesuit Migrants Service, 2024a). Another report shows that there is knowledge about drugs and medical treatment access in Chile for migrants, which is recognized as reasons of migration (Jesuit Migrants Service, 2024b).

## 4 Discussion

As far as we know, this scoping review is the first approximation in Chile to analyze the empirical evidence of scientific articles as well as official reports on migration from 1990 to 2024, with a focus on the period from 2020.

### 4.1 Methodological approaches of the study

From a methodological perspective, the majority of the studies adopted a quantitative, observational, and cross-sectional approach. It can be proven that the main data sources in use corresponded to secondary data (43 % of articles and 68 % of official reports), especially from the CASEN survey and from the Census (Cabieses, 2012b, 2013, 2017; Grande, 2019; Jesuit Migrant Service, 2024a; Margarit, 2022b; National Institute of Statistics, 2018; Oyarte, 2023; Roessler, 2020a, 2020b, 2021b; Roessler, 2021c; Roessler, 2021d, 2022; Vicuña, 2015; Zúñiga, 2022) . This finding suggests how difficult it is to generate primary data that can be used to completely describe and understand the migration phenomenon as well as its complexity. This could be related to the limitations to access the migrant population because of their migration status in the country and their mobility. Similarly, this could be attributed to the dynamic processes of their migration trajectories and to the context of the financing policies for this type of research.

The development of studies is primarily from a perspective of methodological exclusion. This implies the studies have adopted quantitative or qualitative approaches, hindering a comprehensive understanding of the reality of the migration movement in Chile. Therefore, mixed methodologies are necessary to understand migration trajectories, for social interaction, and for settling in the destination country, as well as for the experiences during the process.

The ability to mix methodologies in this research on migration in Chile helps us collect plenty of quantitative data, as well as localize and describe the migrant population living in Chile or specific areas, like the capital or the northern regions. On the other hand, a qualitative approach is useful for collecting information on migrants' strategies to adapt to life in Chile and the factors that make them consider staying here or migrating again, while letting us represent their experiences throughout time.

Mixed methods research helps address several complex events from their causes to the implementation of public policies, with its use depending on the priority or importance given to these approaches to answer the research question, how both methodologies are implemented, when and how the data groups are integrated, and finally, how this data is included in the study on migration (Pérez Peña, 2023).

### 4.2 Subjects of interest of scientific evidence

The labor situation of the population has been highlighted, concerning the main cause of voluntary migration. Furthermore, only 29 % of the analyzed scientific production indicated elements associated with some of the identities of the migrant population (disability, asylum/refuge, African descent, and indigenous origin). However, regarding the sex and gender identities, the studies were limited to separating data per sex from a binary perspective, omitting any evidence of the LGBTIQA+ population. Thus, we can infer that the concept of identity of the mobile population is invisible, linking it to some of the invisible factors that drive migration, such as gender identity discrimination, stigma, criminalization, and other types of violence. Therefore, studies with an intersectional perspective should be promoted, in order to understand how several social categories or stigmas affect the LGBTQA+ migrant population's wellbeing and proper resettling in Chile, and those results should be used to design strategies for reducing the negative effects of such influences.

Due to the above, it is important to take into account the transversal axes of inequalities to which the migrant population may be subject, such as social class, gender, ethnicity/race, and disability, among others, highlighting the subjectivity of their experiences.

### 4.3 Health dimension

The health dimension seems important in the scientific articles (71 %) but not so important in the reports (29 %). This is an interesting finding due to the relevance of the subject. It is concentrated in the access characteristics of healthcare assistance of the migrant population in Chile (Bernales, 2018; Blukacz, 2023; Cabieses, 2012; Cabieses, 2013; Iribarne-Wiff, 2023; Jesuit Migrants Service, 2024a; Oyarte, 2023; Roessler, 2021a, 2022; Rojas, 2011; World Bank, 2022), highlighting access barriers. In this sense, there are initiatives from the Ministry of Health of the Government of Chile to facilitate the administrative procedures. The National Health Fund (FONASA for its acronym in Spanish), a public health insurance, creates a temporary identification number (NIP) assigned to the migrant population, allowing them access to healthcare assistance regardless of their migration status. This is valid for a year. During this period, migrants are supposed to regularize their migration status in the country.

It is confirmed that the migrant population lacks knowledge regarding how the healthcare system in Chile works. This can increase the access barriers and negatively impact the population’s health. For example, having late entries to prenatal care during pregnancy (Cabieses, 2017).

Other concerns regarding the migrant population’s health revolve around MH (Caqueo-Urízar, 2021; Carreño, 2020; Castillo-Lobos, 2023; Chen, 2022; Jesuit Migrants Service, 2024a; Rojas, 2011) indicating that this population (Rojas, 2011) has a high predominance of mental disorders and describing their mental health needs in the context of forced migration (Carreño, 2020). Additionally, there is a focus on comparing the childhood experiences of migrants with those of native people (Caqueo-Urízar, 2021). Lastly, there is a study focusing on the impact of COVID on women from Haiti (Chen, 2022).

### 4.4 South-South migration

All articles and 96 % of the official reports where the migrant population's country of origin could be determined were related to South-South migration - that is, migration from a low- or middle-income country to a similar one (Fernández Castilla, 2005), with just 5 articles and 14 official reports including people from "other countries".

Among the possible factors facilitating migration to Chile are its political stability and economic growth since the return to democracy. The financial crises and the increase of poverty and informal work in their countries of origin, toughened migration laws in developed countries, Chile's proximity and lower cost of travel for Latin American and Caribbean people, and contact networks formed by relatives or friends already living in the country are also factors helping the migrants' arrival to Chile (International Organization for Migration, 2015), which are all reflected in the studies covered by this review.

Regarding South-South migration, migratory behavior should be closely monitored from 2025 on, due to the new immigration policies of the United States of America, which principally affect people from low-income and developing countries (Rodríguez M., 2025).

### 4.5 Strengths and limitations

Our review offers conclusive evidence on the characteristics of scientific articles and official reports addressing migration in Chile after the return to democracy; nonetheless, there are some limitations.

Although we covered the main scientific databases, we may have missed articles from non-indexed journals or non-public official reports, due to the difficulty of searching through alternative institutional ways.

The preferential use of controlled vocabulary, such as MeSH, may have limited the search; however, the range of the search terms helped identify difficult-to-access articles and official reports.

## 5 Conclusions

In conclusion, the evidence concerning migration toward Chile primarily centers on south–south migration, sociodemographic characterization, and challenges related to accessing services, including healthcare. The findings reveal a scarcity of studies that gather primary data, resulting in a lack of relevant indicators to understand aspects such as the causes of migration, attracting factors, migration trajectory, migration status, cross-cultural relationships, or sexual health. Therefore, we recommend employing mixed methodologies to study migrations, incorporating gender, sexual diversity, and intersectional perspectives.

After reading the results, we recommend to explore migrants' transnational practices - especially those regarding their constant and prolonged contact with close relations living in their countries of origin, and how they live with the feeling of belonging to two different places. Also, in order to study the phenomenon from a health or educational perspective, it is essential to identify the key elements of the migrants' social construction of identity and integration, and knowing migrants' levels of segmented assimilation to Chile may be useful. It may also be interesting to know migrants' demand/attraction factors in Chile and supply/expulsion factors in their countries of origin, as the causes of migration -and thus, its consequences- are in both origin and destination.

Lastly, it is essential to have primary information on the different types of violence migrants suffer in their travels and the effects on their sexual, reproductive, and mental health - especially regarding women and sex-gender diversities.

## Funding

This work was supported by the Ministry of Science, Technology, Knowledge and Innovation of the Government of Chile (FONDECYT Regular N° 1220371- COSMIC–Community project-based surveillance of socio-epidemiological aspects linked to sexual health and related communicable diseases in Chile’s migrant population). The funders had no role in the design, data collection and analysis, or in the decision to write and submit this paper for publication.

## CRediT authorship contribution statement

**María Belén Reinoso-Cataldo:** Writing – original draft, Methodology, Formal analysis, Conceptualization, Writing – review & editing, Project administration, Investigation, Data curation. **Valeria Stuardo:** Writing – review & editing, Supervision, Methodology, Conceptualization, Writing – original draft, Project administration, Formal analysis. **Cecilia Bustos-Ibarra:** Writing – original draft, Formal analysis, Writing – review & editing, Supervision, Conceptualization. **Julieta Belmar:** Writing – original draft, Conceptualization, Writing – review & editing, Formal analysis. **Cristian Lisboa:** Writing – original draft, Conceptualization, Writing – review & editing, Formal analysis. **Kenny Low:** Writing – original draft, Conceptualization, Writing – review & editing, Formal analysis. **Sonia Parella Rubio:** Writing – original draft, Conceptualization, Writing – review & editing, Formal analysis. **Constanza Adrian Parra:** Writing – original draft, Conceptualization, Writing – review & editing, Formal analysis. **Mercedes Carrasco-Portiño:** Writing – original draft, Methodology, Formal analysis, Conceptualization, Writing – review & editing, Project administration, Investigation, Data curation.

## Declaration of competing interest

The authors declare that they have no known competing financial interests or personal relationships that could have appeared to influence the work reported in this paper.

## Appendix Supplementary materials

Image, application 1
