# Supplementary material for: Characterization of international migration movements toward Chile: A scoping review of scientific articles and official reports
Source: J Migr Health. 2025 Sep 21;12:100363. doi: 10.1016/j.jmh.2025.100363 (PMC12547016; doi:10.1016/j.jmh.2025.100363)
Supplement: Supplementary file 1 [file mmc1.docx]

**Appendix**

**Appendix A. Preferred Reporting Items for Systematic reviews and Meta-Analyses extension for Scoping Reviews (PRISMA-ScR) Checklist**

| **SECTION** | **ITEM** | **PRISMA-ScR CHECKLIST ITEM** | **REPORTED ON PAGE #** |
| --- | --- | --- | --- |
| **TITLE** | | | |
| Title | 1 | Identify the report as a scoping review. | 1 |
| **ABSTRACT** | | | |
| Structured summary | 2 | Provide a structured summary that includes (as applicable): background, objectives, eligibility criteria, sources of evidence, charting methods, results, and conclusions that relate to the review questions and objectives. | 1 |
| **INTRODUCTION** | | | |
| Rationale | 3 | Describe the rationale for the review in the context of what is already known. Explain why the review questions/objectives lend themselves to a scoping review approach. | 2-4 |
| Objectives | 4 | Provide an explicit statement of the questions and objectives being addressed with reference to their key elements (e.g., population or participants, concepts, and context) or other relevant key elements used to conceptualize the review questions and/or objectives. | 4 |
| **METHODS** | | | |
| Protocol and registration | 5 | Indicate whether a review protocol exists; state if and where it can be accessed (e.g., a Web address); and if available, provide registration information, including the registration number. | 5 |
| Eligibility criteria | 6 | Specify characteristics of the sources of evidence used as eligibility criteria (e.g., years considered, language, and publication status), and provide a rationale. | 6 |
| Information sources* | 7 | Describe all information sources in the search (e.g., databases with dates of coverage and contact with authors to identify additional sources), as well as the date the most recent search was executed. | 5-6 |
| Search | 8 | Present the full electronic search strategy for at least 1 database, including any limits used, such that it could be repeated. | Appendix B and C |
| Selection of sources of evidence† | 9 | State the process for selecting sources of evidence (i.e., screening and eligibility) included in the scoping review. | Figure 1 |
| Data charting process‡ | 10 | Describe the methods of charting data from the included sources of evidence (e.g., calibrated forms or forms that have been tested by the team before their use, and whether data charting was done independently or in duplicate) and any processes for obtaining and confirming data from investigators. | 7  Appendix D and E |
| Data items | 11 | List and define all variables for which data were sought and any assumptions and simplifications made. | Appendix D and E |
| Critical appraisal of individual sources of evidence§ | 12 | If done, provide a rationale for conducting a critical appraisal of included sources of evidence; describe the methods used and how this information was used in any data synthesis (if appropriate). | Not applicable |
| Synthesis of results | 13 | Describe the methods of handling and summarizing the data that were charted. | 7-8 |
| **RESULTS** | | | |
| Selection of sources of evidence | 14 | Give numbers of sources of evidence screened, assessed for eligibility, and included in the review, with reasons for exclusions at each stage, ideally using a flow diagram. | 8  Figure 1 |
| Characteristics of sources of evidence | 15 | For each source of evidence, present characteristics for which data were charted and provide the citations. | 8-9  Table 1-4 |
| Critical appraisal within sources of evidence | 16 | If done, present data on critical appraisal of included sources of evidence (see item 12). | No applicable |
| Results of individual sources of evidence | 17 | For each included source of evidence, present the relevant data that were charted that relate to the review questions and objectives. | 10-21 |
| Synthesis of results | 18 | Summarize and/or present the charting results as they relate to the review questions and objectives. | Table 1 - 4 |
| **DISCUSSION** | | | |
| Summary of evidence | 19 | Summarize the main results (including an overview of concepts, themes, and types of evidence available), link to the review questions and objectives, and consider the relevance to key groups. | 22-25 |
| Limitations | 20 | Discuss the limitations of the scoping review process. | 25-26 |
| Conclusions | 21 | Provide a general interpretation of the results with respect to the review questions and objectives, as well as potential implications and/or next steps. | 26 |
| **FUNDING** | | | |
| Funding | 22 | Describe sources of funding for the included sources of evidence, as well as sources of funding for the scoping review. Describe the role of the funders of the scoping review. | 27 |

JBI = Joanna Briggs Institute; PRISMA-ScR = Preferred Reporting Items for Systematic reviews and Meta-Analyses extension for Scoping Reviews.

* Where *sources of evidence* (see second footnote) are compiled from, such as bibliographic databases, social media platforms, and Web sites.

† A more inclusive/heterogeneous term used to account for the different types of evidence or data sources (e.g., quantitative and/or qualitative research, expert opinion, and policy documents) that may be eligible in a scoping review as opposed to only studies. This is not to be confused with *information sources* (see first footnote).

‡ The frameworks by Arksey and O’Malley (6) and Levac and colleagues (7) and the JBI guidance (4, 5) refer to the process of data extraction in a scoping review as data charting*.*

§ The process of systematically examining research evidence to assess its validity, results, and relevance before using it to inform a decision. This term is used for items 12 and 19 instead of "risk of bias" (which is more applicable to systematic reviews of interventions) to include and acknowledge the various sources of evidence that may be used in a scoping review (e.g., quantitative and/or qualitative research, expert opinion, and policy document).

*From:* Tricco AC, Lillie E, Zarin W, O'Brien KK, Colquhoun H, Levac D, et al. PRISMA Extension for Scoping Reviews (PRISMAScR): Checklist and Explanation. Ann Intern Med. 2018;169:467–473. [doi: 10.7326/M18-0850](http://annals.org/aim/fullarticle/2700389/prisma-extension-scoping-reviews-prisma-scr-checklist-explanation).

**Appendix B: Search strategy syntax for included databases**

| **Database** | **Date of search** | **Search strategy syntax** | **Filters** | **Results** |
| --- | --- | --- | --- | --- |
| Web of Science | 2024/11/04 | (((((TS=((Emigration and Immigration))) OR TS=((Human Migration))) OR TS=((Transients and Migrants))) OR TS=((Population Dynamics))) OR TS=((Freedom of Movement))) AND TS=(Chile) | Publication years: 2024 or 2023 or 2022 or 2021 or 2020 or 2019 or 2018 or 2017 or 2016 or 2015 or 2014 or 2013 or 2012 or 2011 or 2010 or 2009 or 2008 or 2007 or 2007 or 2006 or 2005 or 2004 or 2003 or 2002 or 2001 or 2000 or 1999 or 1998 or 1997 or 1998 or 1997 or 1996 or 1995 or 1994 or 1993 or 1992 or 1991 or 1990; Languages: English or Spanish or Portuguese or French; Document types: Article; Web of Science Index: Science Citation Index Expanded (SCI-EXPENDED) or Social Science Citation Index (SSCI). | 512 |
| Pubmed | 2024/11/04 | (((((Emigration and Immigration[MeSH Terms]) OR (Human Migration[MeSH Terms])) OR (Transients and Migrants[MeSH Terms])) OR (Population Dynamics[MeSH Terms])) OR (Freedom of Movement[MeSH Terms])) ) AND (Chile[MeSH Terms]) | Abstract, Books and Documents, Classical Article, Clinical Study, Clinical Trial, Clinical Trial, Phase I, Clinical Trial, Phase II, Clinical Trial, Phase III, Clinical Trial, Phase IV, Comparative Study, Controlled Clinical Trial, Observational Study, Preprint, Randomized Controlled Trial, Validation Study, English, French, Portuguese, Spanish, Humans, from 1990/1/1 - 2024/11/4. | 198 |
| Scielo | 2024/11/04 | (Migrantes and Chile) (DECS Terms) | Colección: Chile; Idioma: Español; Idioma: Inglés; Año de publicación: 2024-1990; Tipo de literatura: Artículo; WoS Indice de Citaciones: Todos. | 108 |
| Scopus | 2024/11/04 | ( emigration AND immigration ) OR ( human AND migration ) OR ( transients AND migrants ) OR ( population AND dynamics ) OR ( freedom AND of AND movement ) AND chile | ( emigration AND immigration ) OR ( human AND migration ) OR ( transients AND migrants ) OR ( population AND dynamics ) OR ( freedom AND of AND movement ) AND chile AND PUBYEAR > 1989 AND PUBYEAR < 2025 AND ( LIMIT-TO ( DOCTYPE , "ar" ) ) AND ( LIMIT-TO ( LANGUAGE , "English" ) OR LIMIT-TO ( LANGUAGE , "Spanish" ) OR LIMIT-TO ( LANGUAGE , "Portuguese" ) OR LIMIT-TO ( LANGUAGE , "French" ) ) AND ( LIMIT-TO ( SUBJAREA , "SOCI" ) ) AND ( LIMIT-TO ( EXACTKEYWORD , "Article" ) ) AND ( LIMIT-TO ( AFFILCOUNTRY , "Chile" ) ) AND ( LIMIT-TO ( PUBSTAGE , "final" ) ) AND ( LIMIT-TO ( SRCTYPE , "j" ) ) AND ( LIMIT-TO ( PUBYEAR , 1990 ) OR LIMIT-TO ( PUBYEAR , 1991) OR LIMIT-TO ( PUBYEAR , 1992 ) OR LIMIT-TO ( PUBYEAR , 1993 ) OR LIMIT-TO ( PUBYEAR , 1994 ) OR LIMIT-TO ( PUBYEAR , 1995 ) OR LIMIT-TO ( PUBYEAR , 1996 ) OR LIMIT-TO ( PUBYEAR , 1997 ) OR LIMIT-TO ( PUBYEAR , 1998 ) OR LIMIT-TO ( PUBYEAR , 1999 ) OR LIMIT-TO ( PUBYEAR , 2000 ) OR LIMIT-TO ( PUBYEAR , 2001 ) OR LIMIT-TO ( PUBYEAR , 2002 ) OR LIMIT-TO ( PUBYEAR , 2003 ) OR LIMIT-TO ( PUBYEAR , 2004 ) OR LIMIT-TO ( PUBYEAR , 2005 ) OR LIMIT-TO ( PUBYEAR , 2006 ) OR LIMIT-TO ( PUBYEAR , 2007 ) OR LIMIT-TO ( PUBYEAR , 2008 ) OR LIMIT-TO ( PUBYEAR , 2009 ) OR LIMIT-TO ( PUBYEAR , 2010 ) OR LIMIT-TO ( PUBYEAR , 2011 ) OR LIMIT-TO ( PUBYEAR , 2012 ) OR LIMIT-TO ( PUBYEAR , 2013 ) OR LIMIT-TO ( PUBYEAR , 2014 ) OR LIMIT-TO ( PUBYEAR , 2015 ) OR LIMIT-TO ( PUBYEAR , 2016 ) OR LIMIT-TO ( PUBYEAR , 2017 ) OR LIMIT-TO ( PUBYEAR , 2018 ) OR LIMIT-TO ( PUBYEAR , 2019 ) OR LIMIT-TO ( PUBYEAR , 2020 ) OR LIMIT-TO ( PUBYEAR , 2021 ) OR LIMIT-TO ( PUBYEAR , 2022 ) OR LIMIT-TO ( PUBYEAR , 2023 ) OR LIMIT-TO ( PUBYEAR , 2024 ) ) | 81 |

**Appendix C: Information sources of grey literature search**

| **Sources*** | **Link to access the platform of the sources** | **Directions to the report** |
| --- | --- | --- |
| INE | <https://www.ine.gob.cl/> | Click on statistics and click on SOCIAL ITEM: DEMOGRAPHY AND VITALS. Then select DEMOGRAPHY and there are the statistics of the migratory movement. |
| Servicio Nacional de Migraciones | <https://serviciomigraciones.cl/> | Click on MORE and select STUDIES. There you can select by subject: Estimates of foreigners, Open data, SERMIG studies and analysis, Demographic reports,  International studies. |
| Servicio Jesuita a Migrantes | <https://sjmchile.org/> | Click on INCIDENCE AND STUDIES, then PUBLICATIONS. |
| INDH | <https://www.indh.cl/> | Click on Documents, then DIGITAL LIBRARY, click on ENTER, click on SEARCH and type in MIGRATION and the related studies will be displayed. |
| ACNUR | <https://www.acnur.org/> | Click on VIEW MORE DATA AND STATISTICS, then at the bottom of the page click on CONSULT DATA, and here you will find the reports. |

*INE: Instituto Nacional de Estadística (National Institute of Statistics); Servicio Nacional de Migraciones: National Migration Service; Servicio Jesuita a Migrantes (Jesuit Migrant Services); INDH: Instituto Nacional de Derechos Humanos (National Institute of Human Rights); ACNUR: Agencia de la Organización de Naciones Unidas para Refugiados (United Nations Refugee Agency (UNHCR))

**Appendix D: Description of variables for scientific articles**

| **VARIABLES** | **CONCEPTUAL**  **DEFINITION** | | **OPERATIONAL DEFINITION** | |
| --- | --- | --- | --- | --- |
| 1. **Research objective** | The main goal of a particular research. | | General objective | |
| 1. **First author’s name** | Name or denomination given to a specific person. | | First author ‘s name | |
| 1. **Nationality of the first author’s institution** | Country where the institution the first author is affiliated to is. | | Country name | |
| 1. **Year of publication** | Year in which the article was published in a magazine. | | Year of publication | |
| 1. **Journal in which the article is published** | A periodic publication mainly presenting scientific articles, written by different authors, and the latest information on research and development from any and all scientific fields. | | Journal name | |
| 1. **Field of study of the journal** | Field of study the publishing journal is focused on. | | Medicine  Social sciences  Statistics  Epidemiology  Others | |
| 1. **Research methodology** | Set of methods used for approaching a scientific research. | | Quantitative  Qualitative  Mixed | |
| 1. **Study design** | Method(s) used by the researcher to select participants, gather data, analyze it, and interpret the results. | | Type of study design | |
| 1. **Type of source** | Use of primary sources or already existing data for a new purpose. | | Primary data  Secondary data | |
| 1. **Study population** | Group or set of elements intended to be studied or researched on. | Study participants | |  |
| 1. **Study population’s country of origin** | Country where the study population comes from. | Study population’s country of origin | |  |
| 1. **Study population’s sex** | Organic characteristics that allow to distinguish between males and females. | Female  Male  Intersex  Other  Not stated | |  |
| 1. **Study population’s age** | How long a person has lived. | Age | |  |
| 1. **Education level** | The study population’s educational stages. | Years of schooling | |  |
| 1. **Employment** | A person’s main job, including their field and qualification level. | Type of work | |  |
| 1. **Other identities** | Belonging to a human group that shares a status, culture, history, and traditions, and whose members are united by a common identity they identify with. | Disabled  Asylum seekers / Refugees  Descended from African people  Indigenous | |  |
| 1. **Study population’s immigration status** | A migrant’s legal status when entering a country. | Temporary work visa  Permanent work visa  Tourist visa  Irregular | |  |
| 1. **Study population’s type of migration** | How immigration is done, regargding its geographic characteristics and legal status when leaving or entering a specific country. | Domestic  International  Irregular  Regular  Forced / Displaced  Humanitarian migration  Other | |  |
| 1. **Study population’s mother tongue** | A person’s first language, or the main language of a specific nation when talking about people from there. | Study population’s mother tongue | |  |
| 1. **Culture gaps** | Any systematic difference between two cultures that difficults mutual understanding or relations. | Language  Beliefs  Customs  Rites  Practices  Other | |  |
| 1. **Reasons for migration** | The reason(s) why people –temporarily or definitively– leave a country to live in a different one. | Results on the reasons for migration (political, cultural, or socioeconomic factors, as well as problems related to family, healthcare, catastrophes, quality of life, etc.) | |  |
| 1. **Pull factors** | Characterization of the factors drawing people to live in a specific country. | Results on the factors motivating migration to a specific country | |  |
| 1. **Study population’s acculturation** | Process by which a person, group, or people acquires and assimilates the characteristics and elements of a culture other than theirs; a process of integration and/or inclusion to the receiving society. | Results on the study population’s levels of acculturation | |  |
| 1. **Presence of healthcare** | Characteristics of the population’s health, access to healthcare, or another health-related variable. | Description of the study population’s health, access to healthcare, or other health-related variables | |  |
| 1. **Knowledge on the procedures to access healthcare for migrant population** | Migrant population’s level of knowledge on the procedures to access healthcare. | Results on what the study population knows about access to healthcare | |  |

**Appendix E: Description of the variables for official reports.**

| **VARIABLES** | **CONCEPTUAL DEFINITION** | | **OPERATIONAL DEFINITION** |
| --- | --- | --- | --- |
| 1. **Objective of the report** | The main goal of a particular research. | | General objective |
| 1. **First author’s name** | Name or denomination given to a specific person. | | First author ‘s name |
| 1. **Publishing institution** | Institution publishing the report. | | Name of the institution |
| 1. **Type of publishing institution** | The type of organization the institution publishing the report belongs to. | | University  Research center  International organization  National organization  NGO  Foundation  Other |
| 1. **Participing institutions** | All institutions working on the writing and publication of the report. | | Name(s) of the institution(s) |
| 1. **Year of publication** | Year in which the report was published. | | Year of publication |
| 1. **Report writing procedure** | Set of methods used for approaching a scientific research. | | Quantitative  Qualitative  Mixed |
| 1. **Study design** | Method(s) used by the researcher to select participants, gather data, analyze it, and interpret the results. | | Type of study design |
| 1. **Type of source** | Use of primary sources or already existing data for a new purpose. | | Primary data  Secondary data |
| 1. **Study population** | Group or set of elements intended to be studied or researched on. | Study participants | |
| 1. **Study population’s country of origin** | Country where the study population comes from. | Study population’s country of origin | |
| 1. **Study population’s sex** | Organic characteristics that allow to distinguish between males and females. | Female  Male  Intersex  Other  Not stated | |
| 1. **Study population’s age** | How long a person has lived. | Age | |
| 1. **Education level** | The study population’s educational stages. | Years of schooling | |
| 1. **Employment** | A person’s main job, including their field and qualification level. | Type of work | |
| 1. **Other identities** | Belonging to a human group that shares a status, culture, history, and traditions, and whose members are united by a common identity they identify with. | Disabled  Asylum seekers / Refugees  Descended from African people  Indigenous | |
| 1. **Study population’s immigration status** | A migrant’s legal status when entering a country. | Temporary work visa  Permanent work visa  Tourist visa  Irregular | |
| 1. **Study population’s type of migration** | How immigration is done, regargding its geographic characteristics and legal status when leaving or entering a specific country. | Domestic  International  Irregular  Regular  Forced / Displaced  Humanitarian migration  Other | |
| 1. **Study population’s mother tongue** | A person’s first language, or the main language of a specific nation when talking about people from there. | Study population’s mother tongue | |
| 1. **Culture gaps** | Any systematic difference between two cultures that difficults mutual understanding or relations. | Language  Beliefs  Customs  Rites  Practices  Other | |
| 1. **Reasons for migration** | The reason(s) why people –temporarily or definitively– leave a country to live in a different one. | Results on the reasons for migration (political, cultural, or socioeconomic factors, as well as problems related to family, healthcare, catastrophes, quality of life, etc.) | |
| 1. **Pull factors** | Characterization of the factors drawing people to live in a specific country. | Results on the factors motivating migration to a specific country | |
| 1. **Study population’s acculturation** | Process by which a person, group, or people acquires and assimilates the characteristics and elements of a culture other than theirs; a process of integration and/or inclusion to the receiving society. | Results on the study population’s levels of acculturation | |
| 1. **Presence of healthcare** | Characteristics of the population’s health, access to healthcare, or another health-related variable. | Description of the study population’s health, access to healthcare, or other health-related variables | |
| 1. **Knowledge on the procedures to access healthcare for migrant population** | Migrant population’s level of knowledge on the procedures to access healthcare. | Results on what the study population knows about access to healthcare | |
